# Economic Evaluation of Factorial Trials: Cost-Utility Analysis of the Atorvastatin in Factorial With Omega EE90 Risk Reduction in Diabetes 2 × 2 × 2 Factorial Trial of Atorvastatin, Omega-3 Fish Oil, and Action Planning

**DOI:** 10.1016/j.jval.2020.05.018

**Published:** 2020-10

**Authors:** Helen A. Dakin, Andrew Farmer, Alastair M. Gray, Rury R. Holman

**Affiliations:** 1 Health Economics Research Centre, Nuffield Department of Population Health, University of Oxford, Oxford, England, UK; 2 Nuffield Department of Primary Care Health Sciences, University of Oxford, Oxford, England, UK; 3 Diabetes Trials Unit, University of Oxford, Oxford, England, UK

**Keywords:** adherence, economic evaluation, factorial design, individual patient simulation model, lipids, randomized controlled trial, type 2 diabetes

## Abstract

**Objectives:**

We applied principles for conducting economic evaluations of factorial trials to a trial-based economic evaluation of a cluster-randomized 2 × 2 × 2 factorial trial. We assessed the cost-effectiveness of atorvastatin, omega-3 fish oil, and an action-planning leaflet, alone and in combination, from a UK National Health Service perspective.

**Methods:**

The Atorvastatin in Factorial With Omega EE90 Risk Reduction in Diabetes (AFORRD) Trial randomized 800 patients with type 2 diabetes to atorvastatin, omega-3, or their respective placebos and randomized general practices to receive a leaflet-based action-planning intervention designed to improve compliance or standard care. The trial was conducted at 59 UK general practices. Sixteen-week outcomes for each trial participant were extrapolated for 70 years using the United Kingdom Prospective Diabetes Study Outcomes Model v2.01. We analyzed the trial as a 2 × 2 factorial trial (ignoring interactions between action-planning leaflet and medication), as a 2 × 2 × 2 factorial trial (considering all interactions), and ignoring all interactions.

**Results:**

We observed several qualitative interactions for costs and quality-adjusted life-years (QALYs) that changed treatment rankings. However, different approaches to analyzing the factorial design did not change the conclusions. There was a ≥99% chance that atorvastatin is cost-effective and omega-3 is not, at a £20 000/QALY threshold.

**Conclusions:**

Atorvastatin monotherapy was the most cost-effective combination of the 3 trial interventions at a £20 000/QALY threshold. Omega-3 fish oil was not cost-effective, while there was insufficient evidence to draw firm conclusions about action planning. Recently-developed methods for analyzing factorial trials and combining parameter and sampling uncertainty were extended to estimate cost-effectiveness acceptability curves within a 2x2x2 factorial design with model-based extrapolation.

## Highlights

• Methods have been developed for conducting economic evaluations on 2 × 2 factorial trials, but the impact of more complex designs and studies with model-based extrapolation has not yet been explored.
• Atorvastatin has been shown to be cost-effective for patients with type 2 diabetes, whereas omega-3 is not recommended.
• We are not aware of any previous studies exploring interactions between statins, omega-3, and interventions to improve compliance in type 2 diabetes.
• We applied methods for analyzing factorial trials to a trial with a 2 × 2 × 2 design and a model-based extrapolation.
• We observed large interactions between the 3 interventions, although the methods for analyzing the factorial trial did not change the conclusions about which treatment was the best value for the money.
• We extended recent work on combining sampling uncertainty and parameter uncertainty to estimate cost-effectiveness acceptability curves and the value of perfect information.
• We confirmed that atorvastatin is cost-effective, but omega-3 is not.
• Further research on action-planning interventions to improve compliance is likely to be good value for money.

## Introduction

Factorial randomized controlled trials (RCTs) compare different combinations of ≥2 treatments.^1^^,^^2^ These enable ≥2 independent treatments to be evaluated efficiently and allow estimation of interactions between treatments (ie, how much the effect of 1 treatment changes when another treatment is also given). Economic evaluation of factorial trials, however, raises several challenges owing to the inappropriateness of transforming the data and the challenges of regression analysis on health economic data.^2^ Analytical methods are further complicated by more complex factorial designs (eg, those evaluating 3 treatments simultaneously) and the need to extrapolate results beyond the end of the trial; we are not aware of any previous studies that have explored methods for conducting economic evaluations on factorial trials with these features.

Type 2 diabetes is characterized by polypharmacy: in addition to requiring medications to improve glycemic control, guidelines recommend that medication is used to control blood pressure and that both diabetic patients >40 years and younger high-risk groups receive statins to reduce the risk of cardiovascular events.^3^ Atorvastatin is recommended in preference to other statins due to its potency and cost-effectiveness.^4^ Although there is extensive evidence demonstrating that statins are highly effective and cost-effective at reducing low-density lipoprotein cholesterol (LDL-C) levels and preventing cardiovascular events,^5^, ^6^, ^7^, ^8^ there was limited evidence in diabetic patients typical of those managed in primary care at the time the trial was funded.

Omega-3 is not currently recommended^4^; however, there is evidence that it significantly reduces triglycerides and very low-density lipoprotein levels in patients with type 2 diabetes and may raise LDL-C.^9^ Trials in nondiabetic populations have found that it does not significantly reduce the incidence of cardiovascular events.^10^ Many patients do not adhere to diabetes medications,^11^^,^^12^ which reduces the effectiveness of treatment and increases healthcare costs through increased hospitalizations.^12^ Interventions affecting compliance or the likelihood of clinical events may have nonadditive effects on costs and life expectancy.^13^ We are not, however, aware of any previous studies exploring interactions between statins, omega-3, and interventions to improve compliance in patients with type 2 diabetes.

This study aimed to apply the principles for conducting economic evaluations of factorial trials to a cluster-randomized 2 × 2 × 2 factorial trial and to explore the extent to which the results are sensitive to the assumptions made about whether treatments have additive effects. We used the atorvastatin in factorial with omega EE90 risk reduction in diabetes (AFORRD) RCT^14^^,^^15^ to conduct a trial-based economic evaluation assessing the cost-effectiveness of atorvastatin, omega-3 fish oil, and an action-planning leaflet, alone and in combination, in patients with type 2 diabetes from a UK National Health Service (NHS) perspective. We also calculated the expected value of perfect information (EVPI).

## Methods

AFORRD (ISRCTN 76737502) comprised a double-blind RCT conducted in adults with type 2 diabetes and no known cardiovascular events who were managed in UK primary care and were not thought to require immediate lipid-lowering treatment.^14^^,^^15^ The trial used 3 interventions in a 2 × 2 × 2 factorial design.

All 800 patients were simultaneously:

• individually randomized to receive 20 mg/day atorvastatin or matching placebo
• individually randomized to either omega-3 EE90 (Omacor 2 g/day [Pronova BioPharma Norge AS, Lysaker, Norway], containing 46% eicosapentaenoic acid and 38% docosahexaenoic acid; referred to hereafter as omega-3) or matching placebo.

All 59 participating general practices were cluster randomized to either standard care alone or to send patients a paper-based action-planning behavioral intervention intended to increase compliance with study medication (referred to hereafter as “action-planning leaflet”),^15^ alongside standard care. This leaflet was posted to the patient along with other questionnaires 2 weeks after receiving tablets and starting treatment. It comprised 1 A4 sheet that encouraged patients to set and write down an action plan detailing when and where they would take the medication. Patients at the centers randomized to control received only the standard questionnaires.

Patients, therefore, received 1 of 8 combinations: no treatment; atorvastatin; omega-3; action planning; atorvastatin + omega-3; atorvastatin + action planning; omega-3 + action planning; or atorvastatin + omega-3 + action planning.

The protocol was approved by local and national ethics committees and regulatory agencies, and the study was carried out in accordance with the Declaration of Helsinki and good clinical practice guidelines.

We based our economic evaluation solely on an extrapolation of the AFORRD trial (excluding external evidence) to estimate interactions and compare methods for dealing with the factorial design. The analysis took a UK NHS perspective and focused on the costs and clinical endpoints observed over the first 16 weeks of the 1-year trial to compare methods for analyzing a factorial trial, as several aspects of the interventions changed after 16 weeks.^14^^,^^15^ First, all high-risk patients were given an extra 20 mg atorvastatin tablet daily from week 16. Second, research nurses at centers randomized to action planning reinforced patients' action plans face to face at weeks 18 and 32.^14^^,^^15^

Data on compliance with study medication, concomitant medication use, ambulatory consultations, and hospitalizations were collected during the study period and valued using the UK unit costs shown in Appendix Table 1 (in Supplemental Materials found at https://doi.org/10.1016/j.jval.05.018.) Costing analyses used an index year of 2018/19. Study intervention costs were based on 2018/19 prices,^16^, ^17^, ^18^ while older costs for other within-trial resource use and diabetes management in the extrapolated period were adjusted for inflation^17^ (Appendix 1 in Supplemental Materials found at https://dx.doi.org/10.1016/j.jval.05.018).

The within-trial costing analysis included NHS costs accrued in the first 16 weeks after randomization; full details are given in Appendix 1 (in the Supplemental Materials found at https://dx.doi.org/10.1016/j.jval.05.018.) General practitioners (GPs) provided data on concomitant medications at baseline and during the trial, and hospitalizations were identified through adverse event case report forms. Trial participants completed resource use questionnaires providing numbers of consultations with GPs, practice nurses, and outpatient clinics over the full 52-week trial period; these consultation counts were divided by 3.25 (52 ÷ 16) to estimate resource use during the first 16 weeks. Patient EQ-5D utility was adjusted for baseline imbalance and assumed to remain at baseline values during the first 16 weeks (see Appendix 1 in Supplemental Materials found at https://doi.org/10.1016/j.jval.05.018).

To simplify the analysis for this methodological study, the 68 patients with missing 16-week data were omitted from the analysis and conditional mean imputation was used for all remaining missing data.

### Extrapolation and Analysis

We conducted a cost-utility analysis to assess the cost-effectiveness of the 3 interventions in terms of the cost per quality-adjusted life-year (QALY) gained. QALYs capture the effects of cardiovascular events on both length and quality of life. Cardiovascular risk factors (eg, LDL-C and triglycerides) were measured at baseline and at 16 weeks; week 16 data for each trial participant were extrapolated for 70 years using the United Kingdom Prospective Diabetes Study (UKPDS) Outcomes Model version 2.01 (UKPDS-OM2, https://www.dtu.ox.ac.uk/outcomesmodel),^19^, ^20^, ^21^ which predicts the frequency and timing of vascular events and estimates lifetime costs and QALYs. We assumed that body mass index, atrial fibrillation, smoking, high-density lipoprotein cholesterol, LDL-C, blood pressure, and glycated hemoglobin (HbA_1c_) would remain at the values observed at week 16 indefinitely; cholesterol levels have previously been shown to be constant during long-term atorvastatin treatment.^8^ Appendix 1 gives further details on methods for costing, imputation, extrapolation, and analysis (in the Supplemental Materials found at https://doi.org/10.1016/j.jval.05.018).

The UKPDS-OM2 was run with 1 000 000 Monte Carlo loops and 1000 sets of bootstrapped parameters (see Appendix 1 for full details in Supplemental Materials found at https://doi.org/10.1016/j.jval.05.018). We extrapolated data for each individual trial participant as randomized to estimate their lifetime costs and QALYs, as study medications are intended for long-term use and the benefits of lipid-lowering are likely to translate into fewer diabetic events and longer life expectancy.

Within-trial costs and QALYs for each patient were then added to the costs and QALYs extrapolated over a 70-year time horizon. The costs of atorvastatin and omega-3 were based on the level of compliance observed in the first 16 weeks of AFORRD and were assumed to be accrued until death. Action planning was assumed to comprise a one-off intervention received only at baseline. Patients were assumed to adhere fully with concomitant medication for the period in which they took each drug during the 16-week trial, and action planning was assumed to have no impact on concomitant medication costs.

The base case analysis did not adjust for clustering because preliminary analyses using mixed models encountered convergence problems arising from the number of patients per general practice varying between 2 and 55. Although it is generally recommended that 2-stage bootstrapping or multilevel models are used for cluster-randomized trials to allow for between-center variations in how cluster-randomized interventions are administered,^22^^,^^23^ such variations are unlikely within the first 16 weeks of AFORRD because all patients were posted the same leaflet, and no reinforcement was received until after 16 weeks; this was explored in sensitivity analysis.

AFORRD was powered assuming that the 3 interventions would have additive effects on clinical end points, and the factorial design was chosen to address multiple questions without increasing the sample size.^14^ Clinical analyses evaluated each intervention using a different primary end point: the primary end point comprised LDL-C for atorvastatin, triacylglycerol for omega-3, and medication compliance for action plans. Nonetheless, we would expect treatments with multiplicative effects on the incidence of clinical events to have nonadditive effects on long-term costs and QALYs.^2^ Furthermore, we would expect any intervention improving compliance to directly introduce interactions^24^ by increasing the effect of active treatment, but have no impact on outcomes with placebo. Previous trial analyses, however, found that neither omega-3 nor action plans had a significant effect on the primary end points.^14^^,^^15^

We used recently developed methods^25^ to combine parameter uncertainty around UKPDS-OM2 risk equation parameters with uncertainty around the end-of-trial risk factors for the AFORRD sample (described in full in Appendix 1 in Supplementary Materials found at https://dx.doi.org/10.1016/j.jval.05.018). Ten thousand bootstrap samples drawn from the AFORRD trial data set were each extrapolated using one of 1000 sets of UKPDS-OM2 risk equation parameters. Lifetime costs and QALYs for each bootstrap were analyzed using linear regression to adjust for prerandomization values of all UKPDS-OM2 risk factors observed in AFORRD (nonwhite ethnicity, sex, age, duration of diabetes, body mass index, history of atrial fibrillation, smoking, high-density lipoprotein cholesterol, LDL-C, blood pressure, and HbA_1c_). This method also allows for heteroscedasticity and correlations between total lifetime costs and QALYs. Appendix 2 gives a simplified version of the Stata code used (in the Supplemental Materials found at https://doi.org/10.1016/j.jval.05.018).

We compared 3 methods for assessing cost-effectiveness within this 2 × 2 × 2 factorial trial by predicting patients’ lifetime costs and QALYs using linear regression models with 3 different sets of treatment indicators:

1. 2 × 2 (base case): This analysis evaluated the impact of atorvastatin and omega-3 and interactions between these 2 interventions “inside-the-table.”^2^ It ignored the action-planning leaflet because clinical analyses suggested that it had no significant effect on compliance and that compliance was generally high,^15^ implying that the effect on within-trial costs or 16-week cardiovascular risk would be minimal. Regression analyses included dummy variables for atorvastatin allocation, omega-3 allocation, and atorvastatin–omega-3 interaction.
2. 2 × 2 × 2: This analysis took account of all 2- and 3-way interactions among the 3 interventions in the 2 × 2 × 2 design to take account of all interactions and avoid the risk of bias from omitted interaction terms. Regression analyses included 3 main effects and 4 interaction terms.
3. Assuming independence: This analysis estimated the independent effects of atorvastatin, omega-3, and action planning “at-the-margin,”^2^ ignoring all interactions. Because groups were not perfectly balanced, this was analyzed as a single regression with 3 treatment indicators and no interaction terms.

A £20 000/QALY ceiling ratio^26^ was used to calculate net monetary benefit (NMB) and draw conclusions about which treatments are cost-effective. Each of the aforementioned regression models was estimated on each bootstrap replicate. The resulting 10 000 sets of regression coefficients were used to estimate standard errors (SEs), 95% confidence intervals (CIs), *P* values, cost-effectiveness acceptability curves and the EVPI (see Appendix 3 for details). Between 12 and 15 sensitivity analyses varying the methods and assumptions were conducted on each of the 3 analyses (Appendix 3, Tables 6-8 in the Supplemental Materials found at https://doi.org/10.1016/j.jval.05.018.)

Linear regression with robust SEs was used to analyze within-trial costs and resource use, compliance, and change in LDL-C on the original AFORRD sample (without bootstrapping). Such analyses adjusted for baseline levels of all UKPDS-OM2 risk factors, dummy variables for each of the 3 treatments, 3 interaction terms capturing 2-way interactions, and 1 3-way interaction term to avoid bias from omitted interactions and estimate the magnitude of interactions.

All statistical analyses, other than extrapolation in UKPDS-OM2 and estimation of 95% confidence interval (CI) from SEs, were conducted in Stata version 15 (StataCorp, College Station, TX). The opportunity cost of ignoring interactions based on current information^2^ was calculated for analyses 1 and 3 by subtracting the NMB for the treatment that would be adopted within that analysis from the maximum NMB within analysis 2.

## Results

### Within-Trial Findings

All 3 treatments significantly increased the cost of study treatment during the 16-week trial period (*P* < .001; Appendix 3, Table 3 in Supplemental Materials found at https://doi.org/10.1016/j.jval.05.018). There were no statistically significant interactions for study treatment (*P* ≥ .189), although the super-additive (synergistic) interactions between atorvastatin and omega-3 and between omega-3 and action planning were larger than the main effects for atorvastatin and action planning, respectively.

Other resource use and costs during the 16-week randomized trial period were broadly similar across groups, although patients randomized to the action-planning leaflet had significantly fewer ambulatory consultations than those randomized to no action planning (*P* = .049), but with no significant difference in the cost of ambulatory consultations (*P* = 0.105). There were nonsignificant qualitative interactions between action planning and both atorvastatin and omega-3 (ie, nonadditive effects that changed the rankings of treatments with respect to total within-trial cost. Because nonmedication costs varied substantially among patients, atorvastatin (*P* = .749) and action planning (*P* = .398) had no significant effect on total within-trial costs, although omega-3 increased mean total costs by £147.23 (*P* = .023).

No treatments had statistically significant effects on compliance with medication (*P* ≥ .073 when adjusting for all interactions and UKPDS-OM2 risk factors). Patients randomized to atorvastatin monotherapy had significantly greater reductions in LDL-C than those on placebo (mean difference: –1.26, 95% CI, –1.38 to –1.13; *P* < .001), whereas action planning had no significant effect (mean difference: 0.10, 95% CI, –0.03 to 0.23; *P* = .145) and omega-3 monotherapy was associated with a significant *increase* in LDL-C compared with placebo (mean difference: 0.15, 95% CI, 0.02-0.28; *P* = .028). There were, however, statistically significant qualitative interactions that changed the direction of effect for omega-3 (mean interaction between atorvastatin and omega-3, –0.27; 95% CI, –0.45 –0.09; *P* = .004; mean interaction between omega-3 and action planning, –0.23; 95% CI, –0.42 to –0.04; *P* = .019), although no other interactions were significant (*P*≥.070).

### Base Case 2 × 2 Findings

In the 2 × 2 base case analysis (which allowed for interactions between atorvastatin and omega-3 but ignored the action-planning intervention), atorvastatin alone strongly dominated no treatment, saving £262 (95% CI, –£223 to £746; *P* = .288; Table 1) and gaining 0.268 (95% CI, 0.164-0.371; *P* < .0001) QALYs over a lifetime. Adding omega-3 to no treatment had no significant effect on QALYs (*P* = .799) and significantly increased costs by £3884 per patient (95% CI, £3374-£4395; *P* < .0001). There was a nonsignificant qualitative interaction for costs (*P* = 0.205), such that atorvastatin reduced costs when added to no treatment but increased costs when added to omega-3 (Appendix 3, Fig. 2 in Supplemental Materials found at https://dx.doi.org/10.1016/j.jval.05.018). The interaction, however, had no effect on conclusions about which treatment had highest NMB. At a £20 000/QALY ceiling ratio, atorvastatin monotherapy was the most cost-effective treatment, dominating no treatment and omega-3; atorvastatin plus omega-3 cost £192 491/QALY compared with atorvastatin alone.

**Table 1:** Results of the base case lifetime economic evaluation: 2 × 2 analysis of atorvastatin and omega-3 allowing for the interaction between atorvastatin and omega-3.

	Total costs	Quality-adjusted life years	Net monetary benefit∗
No treatment	£29 644 (£582)	10.067 (0.124)	£171 691 (£2116)
Atorvastatin	£29 382 (£562)	10.335 (0.129)	£177 312 (£2245)
Omega-3	£33 528 (£608)	10.076 (0.123)	£167 996 (£2082)
Atorvastatin + omega-3	£33 711 (£605)	10.357 (0.132)	£173 433 (£2254)
Atorvastatin simple effect	−£262 (£247)	0.268 (0.053)†	£5621 (£1039)†
Omega-3 simple effect	£3884 (£260)†	0.009 (0.038)	−£3696 (£764)†
Interaction: atorvastatin by omega-3	£444 (£350)	0.013 (0.054)	−£183 (£1025)

*Note*. Values represent the mean (standard error) for each group for white female nonsmokers without atrial fibrillation who have the mean values for age, duration of diabetes, body mass index, high-density lipoprotein, low-density lipoprotein, blood pressure, and glycated hemoglobin (HbA1c).

∗ Net monetary benefit calculated at a ceiling ratio of £20 000 per quality-adjusted life-year.

† *P* < .05.

### 2 × 2 × 2 Findings

Analysis 2 (controlling for all 3 factors and all interactions in the 2 × 2 × 2 design) estimated broadly similar results to the base case analysis (Table 2; Appendix 3, Fig. 2 in Supplemental Materials found at https://doi.org/10.1016/j.jval.05.018), but found atorvastatin monotherapy to be nonsignificantly more costly than no treatment (*P* = .802). Action planning had very little impact on either costs or QALYs. No interactions were statistically significant (*P* ≥ .082), although the interaction between atorvastatin and action planning was qualitative (ie, larger than either main effect, with the opposite sign) for both costs and QALYs. The 3-way interaction for NMB was qualitative because the simple effect of action planning was so small. Atorvastatin alone cost £2589/QALY compared with atorvastatin plus action planning (the least costly combination), whereas atorvastatin plus omega-3 plus action planning cost £247 097/QALY compared with atorvastatin alone. All other combinations were strongly dominated by atorvastatin with/without action planning (Appendix 3, Fig. 2 in the Supplemental Materials found at https://doi.org/10.1016/j.jval.05.018).

**Table 2:** Results of the lifetime economic evaluation with a 2 × 2 × 2 analysis of atorvastatin, omega-3, and action planning, allowing for all interactions (analysis 2).

	Total costs	Quality-adjusted life years	Net monetary benefit∗
No treatment	£29 388 (£597)	10.053 (0.126)	£171 676 (£2163)
Atorvastatin	£29 462 (£572)	10.366 (0.131)	£177 866 (£2279)
Omega-3	£33 172 (£630)	10.069 (0.126)	£168 205 (£2152)
Action planning	£29 989 (£614)	10.083 (0.128)	£171 674 (£2201)
Atorvastatin + omega-3	£33 555 (£634)	10.336 (0.135)	£173 169 (£2304)
Atorvastatin + action planning	£29 262 (£602)	10.289 (0.134)	£176 524 (£2337)
Omega-3 + action planning	£33 999 (£649)	10.083 (0.127)	£167 662 (£2140)
Atorvastatin + omega-3 + action planning	£33 920 (£629)	10.384 (0.134)	£173 769 (£2277)
Atorvastatin simple effect	£74 (£306)	0.313 (0.060)†	£6190 (£1190)†
Omega-3 simple effect	£3785 (£354)†	0.016 (0.052)	−£3471 (£1066)†
Action-planning simple effect	£601 (£331)	0.030 (0.054)	−£2 (£1052)
Interaction			
Atorvastatin by omega-3	£308 (£482)	−0.046 (0.073)	−£1226 (£1415)
Atorvastatin by action planning	−£801 (£461)	−0.107 (0.077)	−£1340 (£1462)
Omega-3 by action planning	£226 (£510)	−0.016 (0.075)	−£541 (£1478)
Atorvastatin by omega-3 by action planning	£339 (£702)	0.141 (0.103)	£2484 (£1970)

*Note*. Values represent the mean (standard error) for each group for white female nonsmokers without atrial fibrillation who have the mean values for age, duration of diabetes, body mass index, high-density lipoprotein, low-density lipoprotein, blood pressure, and glycated hemoglobin (HbA1c).

∗ Net monetary benefit calculated at a ceiling ratio of £20 000 per quality-adjusted life year.

† *P* < .05.

### Findings Assuming Independence

Analysis 3, controlling for all 3 factors but not allowing for interactions, gave the same conclusions as the other analyses (Table 3). Atorvastatin dominated over no atorvastatin, being significantly more effective (*P* < .0001) and nonsignificantly less costly (*P* = 0.876). Omega-3 cost £255 927/QALY gained compared with no omega-3 and was significantly more costly (*P* < .0001) and had no significant impact on QALYs (*P*=0.531). Action planning cost £119 640/QALY gained, significantly increasing costs (*P* = .028) but with no significant effect on QALYs (*P* = .914).

**Table 3:** Results of the lifetime economic evaluation: assuming independent effects of atorvastatin, omega-3, and action planning, ignoring interactions (analysis 3).

	Total costs	Quality-adjusted life years	Net monetary benefit∗
Atorvastatin	£31 574 (£571)	10.346 (0.129)	£175 348 (£2226)
No atorvastatin	£31609 (£581)	10.072 (0.122)	£169 822 (£2064)
Main effect of atorvastatin	−£34 (£201)	0.275 (0.046)†	£5526 (£919)†
Omega-3	£33 646 (£590)	10.217 (0.125)	£170 691 (£2103)
No omega-3	£29 537 (£558)	10.201 (0.124)	£174 479 (£2119)
Main effect of omega-3	£4109 (£183)†	0.016 (0.026)	−£3788 (£495)†
Action planning	£31 785 (£578)	10.210 (0.125)	£172 423 (£2116)
No action planning	£31 397 (£570)	10.207 (0.124)	£172 747 (£2108)
Main effect of action planning	£388 (£179)†	0.003 (0.027)	−£323 (£512)

*Note*. Values represent the mean (standard error) for each group for white female nonsmokers without atrial fibrillation who have the mean values for age, duration of diabetes, body mass index, high-density lipoprotein, low-density lipoprotein, blood pressure, and glycated hemoglobin (HbA1c).

∗ Net monetary benefit calculated at a ceiling ratio of £20 000 per quality-adjusted life -year.

† *P* < .05.

### Measures of Uncertainty

**Figure 1:**
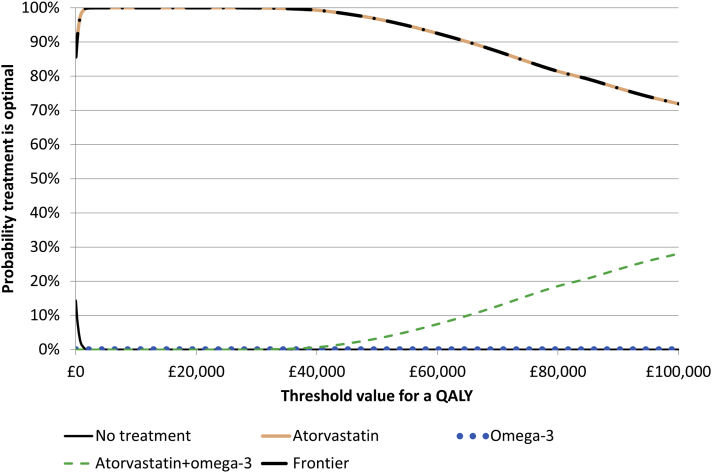
Cost-effectiveness acceptability curves for analysis 1 (base case 2 × 2 accounting for the interaction between atorvastatin and omega-3). QALY indicates quality-adjusted life year.

**Figure 2:**
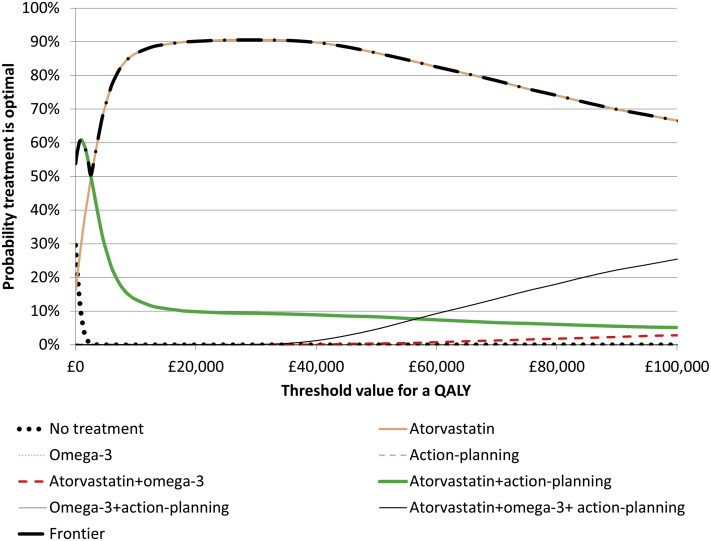
Cost-effectiveness acceptability curves for analysis 2 (2 × 2 × 2 with all interactions). The probability that omega-3, action planning alone or omega-3 + action planning are optimal is ≤0.4% at all ceiling ratios, so these lines lie along the *x* axis in the figure. QALY indicates quality-adjusted life year.

**Figure 3:**
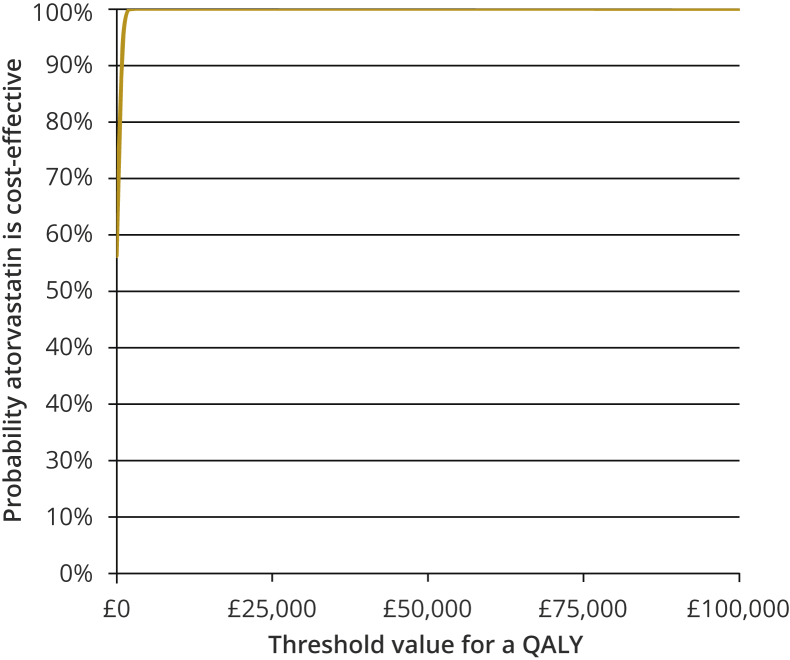
Cost-effectiveness acceptability curve for analysis 3 (assuming independence) for atorvastatin versus no atorvastatin. Curves for the other two comparisons are shown in Appendix 3, Figure 3 in Supplemental Materials found at https://doi.org/10.1016/j.jval.2020.05.018. QALY indicates quality-adjusted life year.

SEs were largest for the 2 × 2 × 2 analysis 2 (considering all interactions) and smallest for analysis 3 (ignoring all interactions). Consequently, the cost-effectiveness acceptability curves rose most sharply when assuming independence and most slowly for 2 × 2 × 2 (Figure 1, Figure 2, Figure 3; Appendix 3, Fig. 3 in Supplemental Materials found at https://doi.org/10.1016/j.jval.05.018). All 3 analyses, however, found that there was a >99.9% chance that atorvastatin (with/without action planning) was the most cost-effective treatment at a £20 000/QALY ceiling ratio. The EVPI was £45/person (£149 million for the United Kingdom over 10 years) for 2 × 2 × 2 (reflecting uncertainty about whether or not action planning is cost-effective), £82/person (£273 million for the United Kingdom) for an independent evaluation of action planning, but £0 for 2 × 2 and for the evaluations of atorvastatin and omega-3 assuming independence (Appendix 3, Table 5 and Fig. 4 in Supplemental Materials found at https://doi.org/10.1016/j.jval.05.018). Because all 3 analyses concluded that atorvastatin with or without action planning had the highest NMB, the opportunity cost of ignoring interactions in analysis 3 was zero at a £20 000/QALY ceiling ratio, although at ceiling ratios between £120 000 and £255 000/QALY, interactions did change the conclusions and ignoring interactions had a substantial opportunity cost (Appendix 3, Fig. 5 in Supplemental Materials found at https://doi.org/10.1016/j.jval.05.018.)

Sensitivity analyses demonstrated that the conclusion that atorvastatin is the most cost-effective treatment was robust to changes in time horizon, compliance to treatment, the reference year for costs, and the price of action planning (Appendix 3, Tables 6-8 in the Supplemental Materials found at https://doi.org/10.1016/j.jval.05.018). When no discounting was applied, atorvastatin plus action planning had the highest expected NMB and a 52% chance of being the most cost-effective treatment. Using a lower discount rate after year 30 did not change the conclusions. Allowing for clustering by general practice, excluding within-trial costs and QALYs and modeling changes in risk factors over time based on recently developed prediction models (Leal et al, written personal communication, March 2019) also had negligible impact on the results. Ignoring the sampling uncertainty around the AFORRD sample reduced SEs by between 1.4% and 7.4%.

Not adjusting for baseline levels of UKPDS-OM2 risk factors was the only sensitivity analysis that changed the conclusion that atorvastatin is cost-effective. When no adjustment was made, omega-3 plus action planning was the most cost-effective treatment within the 2 × 2 × 2 analysis, followed by action planning alone. The unadjusted Analysis 3 (assuming independence) also found action planning to have a >99.9% chance of being cost-effective compared with no action planning (vs 26% in the base case analysis). The conclusions of the 2 × 2 analysis were unchanged, although the probability that atorvastatin alone was the best treatment reduced to 67%.

Analyzing each factor separately (assuming independence and making no adjustment for other factors)^2^ (rather than controlling for all 3 factors within the same regression) found that action planning was less effective than no action planning.

## Conclusions

The results demonstrate that atorvastatin monotherapy is the most cost-effective combination of the 3 treatments evaluated in this 2 × 2 × 2 factorial trial. Neither omega-3 nor action planning significantly increased QALYs, although the 2 × 2 × 2 analysis found that there was a 10% chance that atorvastatin plus action planning is the most cost-effective combination if the NHS is willing and able to pay £20 000 per QALY gained. This confirms previous evidence that atorvastatin is cost-effective as primary prevention for patients with type 2 diabetes,^4^ although a recent review found no good-quality economic evaluations on omega-3 or interventions to improve adherence to statin therapy.^4^ Further research to reduce the uncertainties concerning action-planning interventions may be good value for money, as the EVPI was estimated to be £149 million for the United Kingdom. However, we found that at current prices, further research on atorvastatin or omega-3 would not represent a cost-effective use of public funds.

The results of the sensitivity analysis running regressions separately for each factor highlight the importance of using regression to control for all factors simultaneously,^1^ because even small imbalances in the numbers of patients randomized to the most influential intervention can lead to spurious differences in less influential comparisons unless all factors are controlled for simultaneously.

Although treatment groups appeared balanced at baseline (Appendix 1, Table 2 in the Supplemental Materials found at https://doi.org/10.1016/j.jval.05.018), not controlling for baseline risk factors changed the conclusions of the 2 × 2 × 2 analysis and that assuming independence, highlighting the bias and misleading conclusions that can be introduced by even small baseline imbalances.^25^ Trials with small numbers of patients per arm (such as small factorial trials with large numbers of randomized groups) may be particularly sensitive to bias from baseline imbalance, and cluster-randomized trials may be particularly likely to have baseline imbalance.^22^

This study also provides an example of “ignorable interactions” (ie, those that do not change conclusions about which intervention is the best value for the money).^13^ We observed several qualitative interactions for costs and QALYs, which may have arisen from different cardiovascular interventions having multiplicative effects, the proportional hazards models used within UKPDS-OM2, or from the compliance intervention increasing the effect size for active drugs. These interactions, however, did not change the conclusions at a £20 000/QALY ceiling ratio because atorvastatin is extremely cost-effective, whereas omega-3 is poor value for the money and action planning had little effect on either costs or QALYs. Although many trials are robust to the assumptions about interactions,^27^^,^^28^ the conclusions of others are sensitive to which interactions are included.^29^^,^^30^ The methods for dealing with the factorial design and the assumptions made about interactions are likely to have a larger effect in studies where all treatments affect QALYs and where cost-effectiveness ratios are close to the ceiling ratio.

The conclusions of the economic evaluation should be interpreted with caution because outcomes are extrapolated over a period many times longer than the study period, and we assumed that risk factors, compliance, and treatment effects would remain constant over time. Previous trials suggest that the effect of atorvastatin on lipids is stable over time.^8^ Further research is needed to establish whether any shift in behavior from the action-planning intervention is permanent or whether subsequent interventions are needed to ensure that patients continue (or re-establish) their medication routine over time.

The analysis also ignored uncertainty around the cost and disutility associated with each diabetes-related complication predicted by the model. However, the UKPDS-OM2 takes account of the uncertainty around predictions of cardiovascular events and the importance of interactions is unlikely to be affected by uncertainty around risk factor trajectories or event costs/utilities. Within-trial QALYs could not be accurately assessed because EQ-5D was not measured at 16 weeks. The analysis was based on a single small trial and excludes a substantial body of external evidence demonstrating that statins are effective and cost-effective,^5^, ^6^, ^7^ and that omega-3 does not significantly reduce the incidence of cardiovascular events.^10^

We excluded 68 patients who had missing 16-week data and used conditional mean imputation to impute missing 52-week resource use data because multiple imputation would have substantially complicated the analysis, requiring each patient to be duplicated many times within the UKPDS-OM2, followed by more complicated analyses to combine uncertainty around imputation, extrapolation, and sampling. None of these limitations are likely to have materially influenced the conclusions with respect to the factorial design.

Like 20% of published factorial trial-based economic evaluations,^27^ AFORRD includes cluster randomization. In general, multilevel models or two-stage bootstrap procedures are the most robust methods for dealing with clustering.^23^ We explored multilevel models in a sensitivity analysis and found that clustering by general practice had no impact on the conclusions, probably because the delivery of the cluster-randomized intervention (postal delivery of an action-planning leaflet) is unlikely to have varied between centers during the 16-week period analyzed. Two-stage bootstrapping was infeasible, and some multilevel models converged poorly, as only 40% (23 of 58) of general practices recruited more than 2 patients from each of the 4 individually randomized treatment arms.

This study followed recent recommendations for analyzing factorial trials^2^ and reporting of diabetes models.^31^ The UKPDS-OM2 provided a useful framework for extrapolating data from a 2 × 2 × 2 factorial trial because it simulated lifetime outcomes for individual patients that could be analyzed as-randomized in groups stratified by different factors. Our study extends recent work on handling uncertainty within the UKPDS-OM2,^25^ illustrating the methods in a cluster-randomized factorial trial and by using an additional layer of bootstrapping to estimate cost-effectiveness acceptability curves and the value of perfect information when combining sampling uncertainty and parameter uncertainty.

### Research Data

The UKPDS-OM2 is available at www.dtu.ox.ac.uk/outcomesmodel. A simplified version of the code used to conduct the bootstrapping analyses and control for baseline covariates is available in Appendix 2 in the Supplemental Materials found at https://doi.org/10.1016/j.jval.05.018. Stata code used to run sensitivity analyses is available from the corresponding author on request.
