# Supplementary material for: Economic Evaluation of Factorial Trials: Cost-Utility Analysis of the Atorvastatin in Factorial With Omega EE90 Risk Reduction in Diabetes 2 × 2 × 2 Factorial Trial of Atorvastatin, Omega-3 Fish Oil, and Action Planning
Source: Value Health. 2020 Oct;23(10):1340–8. doi: 10.1016/j.jval.2020.05.018 (PMC7537832; doi:10.1016/j.jval.2020.05.018)
Supplement: Appendix 1 [file mmc1.pdf]

Appendix 1: Additional detail on the methods

The first section provides unit costs and methods for estimating the cost of study medication, while the second section gives additional detail on the methods for estimation of within-trial costs and QALYs.

The remainder of this document gives additional information on the methods used to extrapolate trial results using the UKPDS-OM2 and analyse the model output and follows the structure of the Diabetes Modeling Input Checklist.[1]

Unit costs for within-trial period and cost of study medication

Costing analyses used an index year of 2018-9. List prices[2-4] from 2018-9 were applied for study interventions (Table 1). Unit costs for within-trial resource use other than study interventions were obtained for 2015/16 and inflated to 2017/18 values using Health Services index using the CPI (Health) index.[3] No cost was applied to placebo tablets or capsules since they will not be used in routine clinical practice. Methods for estimating the cost of concomitant medications are described in the next section, while the costs accrued in the extrapolated period are described in the *Costs during the extrapolated period* section, below.

Table 1. Resource use and unit costs accrued during the 16-week trial period

Description	Unit cost 2015-6	Unit cost 2018-9	Source of unit cost
Unit costs obtained June 2019			
20 mg atorvastatin (non-proprietary, per day)	£0.04*	£0.03	£0.81 per 28-tablet pack[2]
2 g omega-3-acid ethyl esters (non-proprietary, per day)	£1.02*	£1.02	£14.24 per 28 1g capsules of eicosapentaenoic acid 460mg / docosahexaenoic acid 380mg capsules.[2] Omacor® has the same unit cost as the non-proprietary formulation.
Action-planning leaflet	£4.53*	£4.32	The leaflet comprised one A4 sheet printed on one side. Within the trial, it was posted to participants along with other questionnaires two weeks after start of treatment. However, for the purposes of the economic evaluation, we assumed that the leaflet would be posted to patients separately using a second-class stamp and enclosing a second stamped addressed envelope for the patient to return their action-plan. The leaflet intervention was therefore assumed to cost £4.32: the price of two second-class stamps from March 2019 (£1.22[4]), plus an estimate of the price of the two envelopes and printing (£0.10) and 10 minutes for a GP administration/clerical worker (£3.00[3]). The cost of the leaflet was assumed to be incurred regardless of what if any active treatment patients were receiving.

**Description**	**Unit cost 2015-6**	**Unit cost 2018-9**	**Source of unit cost**
Unit costs obtained 2015-16 and inflated to 2018-9 values using the Health Services index that was calculated using the Consumer Prices Index (CPI) Health index[3]			
GP home visit	£83.58	£86.11†	Cost per home visit lasting 23.4 minutes, including 11.4 minutes with the patient and 12 minutes travel time, valued at £3.50 per minute of patient contact, including qualification costs, and travel, but excludes direct staff costs (since these are captured in nurse visits).[5] The cost of three miles' travel at 56p per mile is added (the rate given on page 174 for nurses; 3 miles is assumption, which is consistent with the travel cost of £1.50 in 2010[6]).
GP consultation	£40.00	£41.21†	Cost per home visit lasting 11.7 minutes, including qualification costs, and travel, but excludes direct staff costs (since these are captured in nurse visits).[5]
GP telephone consultation	£25.00	£25.76†	Cost per telephone consultation lasting 7.1 minutes (mean length of phone consultation): includes qualification costs, but excludes direct staff costs since nurse visits are costed separately.
Nurse home visit	£27.35	£28.17†	Cost per hour of face-to-face contact (£56 including qualifications) for a GP practice nurse, multiplied by the mean length of the average nurse visit (15.5 minutes), plus the average travel time for GP home visits.[5] Added cost of 3 miles' travel at £0.56/mile (3 miles is assumption, which is consistent with the travel cost of £1.50 in 2010[6]).
Nurse consultation	£14.47	£14.90†	Cost per hour of face-to-face contact (£56 including qualifications) for a GP practice nurse, multiplied by the mean length of GP nurse contacts (15.5 minutes).[5]
Nurse telephone consultation	£6.63	£6.83†	Cost per hour of face-to-face contact (£56 including qualifications) for a GP practice nurse, multiplied by the mean length of GP phone consultation (7.1 minutes).[5]
District nurse home visit	£37.26	£38.39†	Assumed to be the same as a standard district nurse consultation, since district nurses most commonly do home visits.
District nurse consultation	£37.26	£38.39†	National average cost for District Nurse, Adult, Face to face, One to One[7]
District nurse telephone consultation	£16.53	£17.03†	National average cost for District Nurse, Adult, Non face to face[7]
Dietician home visit	£82.66	£85.17†	Assumed to be the same as a standard dietician consultation
Dietician consultation	£82.66	£85.17†	National average cost for dietician consultation.[7]
Dietician telephone consultation	£51.66	£53.23†	Assumed to cost 63% of the price of a surgery consultation, since GP telephone consultations cost 63% of a surgery consultation
Chiropodist home visit	£39.63	£40.83†	Assumed to be the same as a standard podiatry consultation
Chiropodist consultation	£39.63	£40.83†	National average cost for Podiatrist, Tier 1, General podiatry, A09A[7]
Chiropodist telephone consultation	£24.77	£25.52†	Assumed to cost 63% of the price of a surgery consultation, since GP telephone consultations cost 63% of a surgery consultation
Optician visit	£21.31	£21.96†	The NHS sight test fee from 1st April 2015 to 31st March 2016[8]
Telephone optician	£0.00	£0.00	Applied no cost based on the assumption that high-street opticians will not be able to claim NHS payments for telephone consultations.
Hospital Eye clinic visit	£88.84	£91.53†	Weighted average of all non-admitted face-to-face outpatient attendances for "Ophthalmology", "Medical Ophthalmology",

Description	Unit cost 2015-6	Unit cost 2018-9	Source of unit cost
			"Orthoptics" or "Optometry": first and follow-up, and single and multi-professional visits[7]
Telephone hospital eye clinic	£67.88	£69.93†	Weighted average of all non-admitted non-face-to-face outpatient attendances for "Ophthalmology", "Medical Ophthalmology", "Orthoptics" or "Optometry": first and follow-up, but excluding multi-professional visits[7]
Outpatient visit	£110.20	£113.54†	Weighted average of all non-admitted face-to-face outpatient attendances outside of paediatric or child/adolescent departments: both consultant-led and non-consultant led and first and follow-up[7]
Telephone outpatient	£65.68	£67.67†	Weighted average of all non-admitted face-to-face outpatient attendances outside of paediatric or child/adolescent departments: both consultant-led and non-consultant led and first and follow-up, but excluding multi-professional visits[7]
Hospitalisation	£3,178	£3274†	Costs for 18 individual hospitalisations were obtained from 2014-15 Department of Health reference costs[7] by taking a weighted average across the cost per bed day of all healthcare resource with keywords, such as "cataract". All surgical admissions (e.g. hip replacement or pacemaker insertion) were assumed to be elective, while all other admissions (e.g. chest pain) were assumed to be non-elective. Values were inflated to 2018-9 values using Health Services index using the CPI (Health) index.[3] For each admission, we assigned the average cost of a hospital stay across that set of HRGs. For hospital stays longer than the "trim point" defined in the NHS HRG grouper, we applied the cost per hospital stay, plus the excess bed day cost for any bed days beyond the trim point. The figure shown in this row represents the average across all 18 hospitalisations and is given for information only (the costs for each individual hospitalisation were assigned to the relevant patient within the analysis).

* Obtained from earlier versions of the same references used to obtain 2019 prices. Used only in sensitivity analysis

† Inflated from 2015-16 values using the Health Services index that was calculated using the Consumer Prices Index (CPI) Health index.[3]

Compliance with study medication was monitored during the trial using an electronic medication monitoring device (eMems V®, Aardex, Switzerland), which registers the date and time every time the medicine bottle is opened; in line with previous analyses, we assumed that each bottle opening represents medication intake.[9] The base case analysis used the number of tablets/capsules taken to estimate the cost of statin and omega-3. We took the data on the percentage compliance for tablets and for capsules for each patient during the first 16 weeks of the study and multiplied these percentages by the daily cost for statins and omega-3, respectively, to get daily medication costs for each patient. In the absence of any data on how compliance might change over time, patients were assumed to continue at the same level of compliance observed in the 16-week trial for the remainder of their lifetime.

Compliance data were missing for 51 patients (7%) included in the economic evaluation; for the patients with missing data, we applied the mean compliance for each patient's treatment group, but assumed that allocation to active statin treatment had no effect on compliance with omega-3 capsules and vice versa. For example, compliance with omega-3 capsules was assumed to be 81.3% for the 17 patients in the group randomised to no leaflet and active omega-3 who were missing compliance data, based on the average across the 200 patients across the treatment group receiving atorvastatin, omega-3 and no leaflet, and the group receiving no atorvastatin, omega-3 and no leaflet.

Additional methods for estimating within-trial costs and QALYs

Data on ambulatory consultations after randomisation were missing for 122 patients who attended the Week 16 visit but did not complete the 52-week resource use questionnaire, of whom 106 patients completed a questionnaire describing their resource use in the year before randomisation. For these 106 patients, we calculated the cost of consultations attended by each patient in the year before enrolment and added on the mean change in consultation costs between the baseline and 52-week questionnaires in that patient's 2x2x2 study arm. For the 16 patients who were missing resource use questionnaires at both baseline and week 52, we applied the overall mean consultation costs for that patient's 2x2x2 study arm. Missing data on individual questions was imputed as zero based on the assumption that patients would have entered a non-zero value if they had visited this type of healthcare professional.

Participants' GPs provided details of concomitant medication at baseline and reported all changes in concomitant medication occurring during the trial. We calculated the number of days for which each medication was prescribed during the first 16 weeks of the trial.

The within-trial costing analysis included the cost of all medications. However, medications used to treat diabetes and diabetes-related complications (e.g. cardiovascular disease) were valued based on the cost per day for the specific drug and dose prescribed, whereas the cost of all unrelated medications was based on the average cost per prescription across all drugs in the same chapter of the British National Formulary.[10] Participants' GPs reported the reason for each prescription (e.g. "diabetes mellitus" or "asthma"), which we used to identify which prescriptions were related to diabetes. Judgements about which reasons for prescriptions were related to diabetes and diabetes-related complications were made by a clinician. All prescriptions for cardiovascular disease, hypertension, neuropathy, deep-vein thrombosis, microalbuminuria, amyotrophy were considered

diabetes-related. Prescriptions for skin conditions, erectile dysfunction, weight gain/loss and non-diabetes endocrine disorders were considered unrelated. Prescriptions for oedema/swollen ankles, shortness of breath and chest pain were examined on a case-by-case basis to assess whether they were related to cardiovascular disease. No cost was assigned to statins or omega-3 capsules (since these should not have been prescribed alongside study medication), or garlic, evening primrose oil and over-the-counter medications (since these would not normally be prescribed on the NHS).

If the duration of treatment was less than 28 days, we assigned the total cost per prescription (rather than applying a cost per day): for example, we assigned the average cost of prescriptions in BNF chapter 5 for a seven-day course of antibiotics. If the duration of treatment was more than 28 days, we calculated the cost of that medication during the 16-week trial by multiplying the cost per day (for that BNF chapter or for that drug dose) by the number of days of treatment. Diabetes medications were costed using the generic formulation (if any) if the generic drug name was entered, and were assumed to be prescribed as tablets rather than capsules or other formulation). If the brand name was given, it was assumed that that branded drug was prescribed. Drugs prescribed “PRN” were assumed to be used once per day; those prescribed “four or more times daily” were assumed to be given four times per day; those prescribed “monthly” were assumed to be given once every 28 days.

Trial participants completed EQ-5D-3L questionnaires at baseline and 52 weeks. The UK time trade-off tariff[11] was used to estimate utilities based on patients’ questionnaire responses. Outcomes at 52 weeks were not included in QALY calculations, since these could be affected by the addition of atorvastatin and/or behavioural reinforcement beyond Week 16. Within-trial QALYs for each patient were calculated by multiplying patients' baseline utility by 16/52, then centring by subtracting the mean QALYs for the relevant cell of the 2x2x2 factorial design and adding on the grand mean across the whole sample. Within-trial QALYs were assumed to equal the grand mean for the 36 patients with missing baseline EQ-5D utility. This centring method removes any baseline imbalance in EQ-5D utilities, while ensuring that within-trial QALYs vary between patients and that the analysis uses a 40.3-year time horizon for both QALYs and costs. However, it means that all study arms have the same mean QALYs during the 16-week within-trial period.

Methods for extrapolating outcomes and analysing results

Simulation cohort

We extrapolated 16-week data for each of the 732 AFORRD participants who attended the 16-week visit. This avoided the need to simulate hypothetical patients from parametric distributions.

The 68 patients missing all 16-week data were omitted from the analysis to simplify the analysis and focus on methods for dealing with the factorial design. Multiple imputation of missing data would have reduced the potential for bias if data on these patients were missing at random (rather than missing completely at random), but would not have reduced the size of standard errors (SEs) since uncertainty around imputed values would have needed to be propagated through subsequent analyses.

One patient had missing data on HbA1c at the screening visit, although their HbA1c was recorded at week 16. For this patient, we imputed the baseline HbA1c assuming that their HbA1c at the screening visit is equal to their HbA1c at week 16, minus the average change in HbA1c between screening and Week 16 that was observed in the group of patients randomised to the same combination of treatments. This imputed value was not used in the UKPDS-OM2 (where we extrapolated 16-week data), but was used as a pre-randomisation value when adjusting for baseline imbalance. One patient of Chinese ethnicity was coded as “white” within the UKPDS-OM2, since the model does not account for this category separately.

Baseline characteristics of the 732 patients included in the economic evaluation are shown in Table 2; other baseline characteristics have been reported previously for the whole sample.[12] AFORRD recruited patients with no known cardiovascular events not thought by their general practitioner to be at high enough cardiovascular disease risk to require immediate lipid-lowering therapy. Patients who were taking lipid-lowering treatment at baseline were excluded from the trial. During the 16-week trial period, 98% (721/732) of patients had at least one prescription for non-study medication; these included glucose lowering treatments, insulins, aspirin and antihypertensives. Data on physical activity were not collected in the trial.

Table 2. Baseline characteristics for the eight groups. Values represent means (standard deviations) for continuous endpoints and percentage (n/N) for binary endpoints.

Treatment group†	0	A00	0F0	AF0	00L	A0L	0FL	AFL	p‡
Total no. patients in the economic evaluation	105	106	107	73	110	77	76	78	N/A
White ethnicity	14.3% (15/105)	9.4% (10/106)	12.1% (13/107)	4.1% (3/73)	10.0% (11/110)	3.9% (3/77)	2.6% (2/76)	5.1% (4/78)	0.031*
Female	58.1% (61/105)	67.0% (71/106)	61.7% (66/107)	54.8% (40/73)	54.5% (60/110)	58.4% (45/77)	51.3% (39/76)	55.1% (43/78)	0.470
Age at diagnosis of diabetes (years)	66.1 (11.5)	63.5 (11.4)	65.3 (12.5)	61.8 (11.0)	64.4 (12.3)	63.3 (12.1)	60.5 (11.3)	63.1 (10.6)	0.037*
Duration of diabetes (years)	6.8 (6.1)	6.7 (6.7)	5.4 (5.2)	5.3 (4.3)	5.4 (5.7)	6.2 (5.3)	5.0 (4.7)	6.3 (6.1)	0.224
Weight (kilograms)	85.8 (17.7)	88.3 (19.7)	86.8 (16.2)	89.4 (20.2)	86.5 (20.7)	88.7 (17.7)	90.4 (21.4)	89.2 (20.9)	0.727
Height (metres)	1.69 (0.09)	1.70 (0.10)	1.69 (0.09)	1.69 (0.09)	1.67 (0.10)	1.69 (0.11)	1.70 (0.10)	1.69 (0.09)	0.643
Body mass index (kg/m^2)	30.1 (6.0)	30.4 (6.2)	30.5 (5.4)	31.2 (5.8)	30.8 (6.4)	31.2 (5.9)	31.6 (7.8)	31.1 (6.9)	0.775
Atrial fibrillation at screening	2.86% (3/105)	1.89% (2/106)	0.0% (0/107)	0.0% (0/73)	0.0% (0/110)	1.3% (1/77)	1.32% (1/76)	1.28% (1/78)	0.435
Current smoker	6.7% (7/105)	14.2% (15/106)	16.8% (18/107)	15.1% (11/73)	10.0% (11/110)	13.0% (10/77)	10.5% (8/76)	7.7% (6/78)	0.297
High-density lipoprotein at screening (mmol/L)	1.16 (0.30)	1.18 (0.26)	1.16 (0.27)	1.14 (0.32)	1.16 (0.27)	1.12 (0.23)	1.16 (0.29)	1.15 (0.29)	0.952
Low-density lipoprotein at screening (mmol/L)	2.96 (0.74)	3.17 (0.76)	3.09 (0.72)	3.41 (0.77)	3.19 (0.78)	3.19 (0.69)	3.11 (0.71)	3.35 (0.72)	0.002*
Systolic blood pressure at screening (mmHg)	145.4 (16.8)	142.8 (17.3)	140.7 (16.6)	145.1 (15.2)	141.2 (15.9)	144.8 (16.1)	140.9 (14.2)	142.5 (17.2)	0.240
Glycated haemoglobin (HbA1c) at screening (%)	6.84 (1.01)	7.02 (1.04)	7.08 (1.17)	6.86 (1.08)	6.84 (1.09)	7.13 (1.05)	6.87 (1.08)	6.90 (1.06)	0.381
Baseline 10-year coronary heart disease risk (%)‡	23.9 (15.8)	24.0 (16.0)	24.2 (15.1)	22.4 (14.5)	22.7 (15.5)	24.2 (14.8)	18.0 (13.6)	22.6 (14.7)	0.170
Baseline 10-year cardiovascular risk (%)‡	34.6 (18.2)	33.6 (16.9)	34.0 (15.9)	31.9 (16.5)	32.3 (16.9)	34.2 (17.0)	27.2 (15.7)	32.0 (16.6)	0.126

* p<0.05
†Treatment is coded as atorvastatin (A or 0), omega-3 fish oil (F or 0), action-planning leaflet (L or 0).
‡ Global tests for differences between groups, based on regression analyses with three main effects and four interactions (F test based on OLS for continuous endpoints and chi-squared test based on logistic regression for binary endpoints). Omega-3*leaflet and three-way interactions were omitted from the analysis on atrial fibrillation to enable convergence.
‡ Calculated using the UKPDS risk engine.[13,14] Based on 731 patients, excluding one patient who was missing baseline HbA1c.

Heart rate, white blood cell count, haemoglobin and estimated glomerular filtration rate (eGFR) were not recorded in AFORRD; we therefore set these risk factors to the mean values observed in

the Lipids in Diabetes study[15] for all patients (72 bpm, 6.8x10^9/L, 14.5 g/dL and 77.5 mL/minute/1.73 m^2, respectively). The eGFR value that we assumed in the model is consistent with the fact that patients with creatinine ≥181 µmol/L (equivalent to eGFR ≤39 mL/minute/173 m^2) were excluded from AFORRD and is well above the range of eGFR values (<60 mL/minute/173 m^2) thatare a strong predictor of complications in the UKPDS-OM2 version 2.

Since patients with existing cardiovascular events (previous myocardial infarction, established coronary heart disease or other macrovascular disease) were excluded from the trial and none of the events captured in UKPDS-OM2 occurred during the first 16 weeks of the trial, we assumed that no patients had albuminuria, peripheral vascular disease, ischaemic heart disease, heart failure, amputation, stroke or myocardial infarction at the start of the simulation. We also assumed that no patients had renal failure, foot ulcers or blindness at baseline, in the absence of any evidence to the contrary.

No subgroup analyses were done since the current paper focused on methodological principles and as each of the eight treatment groups contained, on average, fewer than 100 patients.

Treatment

Patients were individually randomised to receive 20 mg atorvastatin or matching placebo, and to either omega-3 EE90 (Omacor 2 g/day) or matching placebo. GPs were also cluster-randomised to send patients a paper-based action-planning intervention or standard care. No cost was assigned to placebo, since it was assumed to be equivalent to no treatment. These treatments were an addition to any treatment that patients were already receiving.

Patients were assumed to continue the same study medication until death, with no switching or treatment escalation. As described in *Unit costs for within-trial period and cost of study medication*, compliance was assumed to remain at the level observed in Weeks 1-16 throughout the extrapolated period.

The linkages between risk factors and clinical events, costs and QALYs that are built into the UKPDS-OM2 have been described previously.[15]

The effect of treatment on risk factors was based on the risk factor values observed for each trial participant 16 weeks after the start of randomised treatment. Sixteen-week data for each of the 732

trial participants were entered into the UKPDS-OM2 and extrapolated for 70 years. This approach captures nonlinear relationships between risk factors and outcomes and avoids the need to simulate risk factor data. This ensures that the data capture correlations between risk factors and treatment indicators that are present within real-world data but may be difficult to simulate realistically.

The base case analysis assumed that all risk factors (other than age and event history, which are automatically updated within the UKPDS-OM2) would remain constant over the 70-year extrapolated period, based on the assumption that doses of concomitant medication will be adjusted to maintain risk. However, a sensitivity analysis used the risk factor prediction equations developed for the UKPDS-OM2 (see Appendix 3, *Sensitivity analysis* section). We assumed that the end of trial risk factors explain all differences in clinical events and mortality.

Costs during the extrapolated period

Costs in the absence of complications and the costs associated with diabetic events were based on the default values in UKPDS-OM2,[16] adjusted only for inflation. The diabetic events for which costs were assigned comprised: ischaemic heart disease, myocardial infarction, heart failure, stroke, amputation, blindness, renal failure and ulcer. As per the default costs built into the UKPDS-OM2, costs were assumed to vary with age (in 10-year age bands) and separate costs were used for fatal events, non-fatal events in the year in which the event occurred and for subsequent years.

We used the “currency conversion value” input within the UKPDS-OM2 to inflate costs from 2011/2 values to 2018/19 values using the Health Services index calculated using the Consumer Price Index (CPI) Health index.[3] The 2011/2 costs have been reported previously alongside the methods, unit costs and resources quantities.[16]

The cost of study medication was based on the cost of atorvastatin and omega-3, adjusted for the level of adherence observed for each patient in the 16-week trial (see *Unit costs and cost of study medication* section of this appendix, above). Lifetime costs were estimated from discounted life expectancy after the UKPDS-OM2 simulation had been run (see *Analysis of UKPDS-OM2 output* section of this appendix, below).

Costs were discounted at 3.5% per annum, following recommendations by the National Institute for Health and Care Excellence (NICE).[17] No discounting was applied to the four-month trial period or the first 12 months of the simulation.

Health state utilities

The initial utility for the cohort (0.807) and the utility reductions associated with diabetic events were based on the default values in UKPDS-OM2.[18] These values were based on EQ-5D-3L questionnaires completed by UKPDS trial participants and valued using the UK time trade-off tariff.[11] Utility decrements were assigned for myocardial infarction (-0.065), heart failure (-0.101), stroke (-0.165), amputation (-0.172), renal failure (-0.330) and ulcer (-0.210)[18]; with the exception of myocardial infarction (which was assumed to have no utility decrement in subsequent years), the utility decrement in subsequent years was assumed to be the same as the year when the event occurs. These values and their methods have been reported previously.[18]

Utility decrements were applied in the default way used in the UKPDS-OM2.[19] Events were assumed to have additive effects on utility, with each event reducing utility by a fixed amount regardless of whether any different events have occurred. However, each utility decrement for subsequent years was applied only once: for example, patients who have had two strokes were assumed to have the same utility as those who have had one stroke, whereas patients who have had a stroke and an amputation were assumed to have the decrements for both events.

QALYs and life-years were discounted at 3.5% per annum, following NICE guidance.[17] No discounting was applied to the four-month trial period or the first 12 months of the simulation.

General model characteristics

Trial data were extrapolated using the UKPDS-OM2 version 2.01b5. The model structure, assumptions and data inputs underpinning this model have been reported previously.[15,19] Risk equations predicting the risk of complications and mortality with/without events were estimated using the 5,102 patients participating in the UKPDS trial, who were followed up for a median of 17.6 years.[15]

UKPDS-OM2 is a patient-level simulation model and uses Monte Carlo simulation to simulate the occurrence of eight diabetes-related complications (myocardial infarction, stroke, ischaemic heart disease, congestive heart failure, amputation, blindness, renal failure and ulcer) based on individual patient characteristics and event history.[15]

Since it is a patient-level simulation model, UKPDS-OM2 simulates hypothetical medical histories or "loops" for each trial participant, in which that individual may experience specific events at certain times and will accrue a particular life expectancy. Large numbers of loops must be run to minimise Monte Carlo error and give accurate results.[19,20] The base case analysis was based on 1 million inner 'loops' to ensure that Monte Carlo error was negligible. We conducted preliminary analyses assessing how SEs vary with the number of loops, which suggested that SEs for the difference between atorvastatin and no atorvastatin in Analysis 3 (assuming independence) differ very little between 1000 loops and 1 million loops, but that Monte Carlo error has a larger effect on SEs when sample sizes are smaller. We used the largest number of loops that generated results in a feasible length of time since one of the primary aims of this paper was to compare SEs between Analyses 1, 2 and 3 and as the number of patients per treatment arm was considerably smaller (between 73 and 110) in Analysis 2. For practical reasons, we ran the model in five batches, with each batch using a different set of 200 bootstraps and different random number seeds but all forecasting outcomes for all 732 patients. This enabled the analyses to be run in parallel on different virtual computers, thereby generating results more quickly. Sensitivity analyses that required rerunning the UKPDS-OM2 used 5000 loops in order to generate results in a feasible length of time. The main analysis took 19.5 days to simulate on two 10-core simulation servers, while each sensitivity analysis took between 2.1 and 11.3 hours.

UKPDS-OM2 captures parameter uncertainty around risk equation parameters by providing 5000 sets of risk equation coefficients that were estimated by bootstrapping the original UKPDS sample; these bootstraps allow for correlations between different risk equations and between coefficients from the same equation.[15,19] We used 1000 set of bootstrapped parameters, since preliminary analyses suggested that 800 bootstraps was sufficient to estimate SEs to ±10% accuracy based on the methods of O'Hagan et al[21] and gave stable results (Fig. 1).

Fig. 1. Convergence of (A) incremental costs and (B) incremental QALYs for atorvastatin alone vs. no treatment in Analysis 1 across the 1,000 sets of UKPDS model parameters

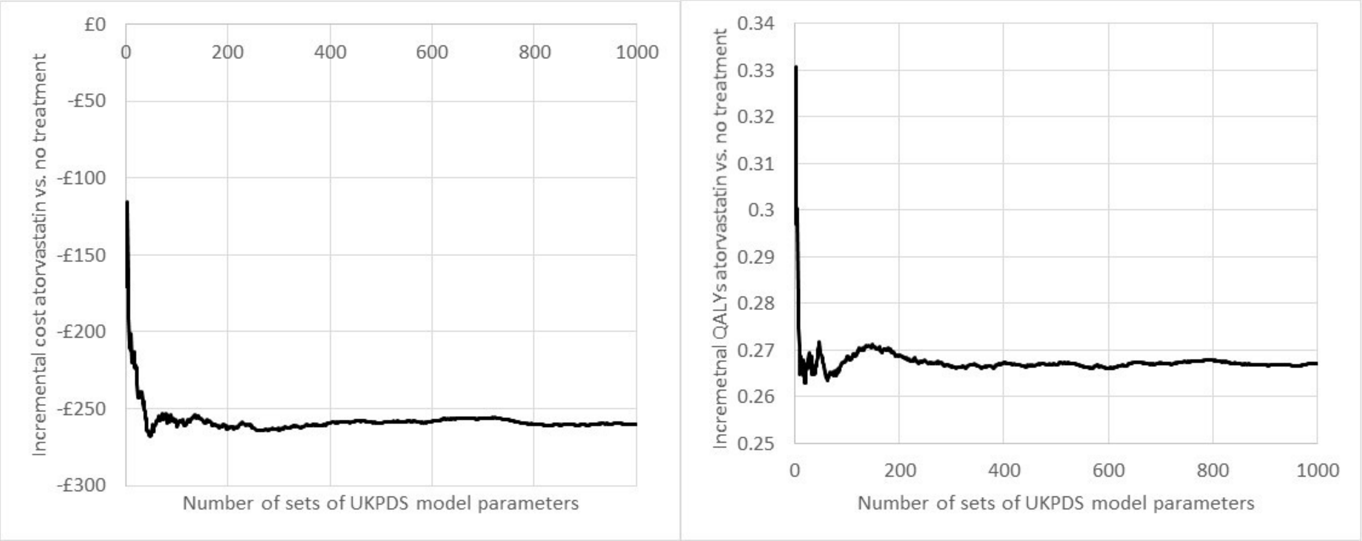

Results represent the cumulative mean coefficient for atorvastatin for the original AFORRD sample (without bootstrapping) across the 1000 sets of UK PDS model parameters.

We accounted for sampling uncertainty around treatment effects and post-treatment risk factor levels by extrapolating data for individual trial participants. Uncertainty around the assumption of constant risk factors was not included in SEs, 95% CI or cost-effectiveness acceptability curves, although the assumption of constant risk factors was relaxed in a sensitivity analysis. Uncertainty around current treatment adherence was captured by using the level of adherence observed for each individual trial participant; however, the analysis did not allow for uncertainty around the assumption that compliance would not change over time. Uncertainty around the utility decrements and costs applied to each diabetic complication was not captured in the analysis since the functionality to do so is not yet built into the UKPDS-OM2. Uncertainty around missing data at the 16-week follow-up was not captured in the analysis. Sufficient loops were run to ensure that stochastic uncertainty (Monte Carlo error) was negligible.

The methods described in the *Analysis of UKPDS-OM2 output* section below were used to ensure that uncertainty around both UKPDS-OM2 risk equation parameters and risk factors within the AFORRD sample was captured in cost-effectiveness acceptability curves and SEs.

Aspects of methodological and structural uncertainty were explored in the sensitivity analyses described in the *Sensitivity analysis* section of Appendix 3.

The discounting start year was set to zero for all patients to ensure that discounting was not applied until the second year of simulated data.

Analysis of UKPDS-OM2 output

The UKPDS-OM2 estimates lifetime costs and QALYs for M sets of bootstrapped risk equation parameters. The beta version of the UKPDS-OM2 (version 2.01b5) we used has the option to output a matrix containing the lifetime costs, QALYs and life years for each bootstrap of each patient. This functionality is now available to all users in version 2.1, where a .csv file containing this matrix is saved when the “Patient bootstraps” option is selected. We used this matrix to conduct a number of post-simulation processing steps and estimate the effect of treatment and between-treatment interactions while combining sampling and parameter uncertainty, allowing for covariates, clustering by GP practice and correlations between costs and QALYs.

The UKPDS-OM2 version 2.01b5 was set up to handle up to three treatment groups, each with fixed annual therapy costs that do not vary between patients within that group. By contrast, for this study we needed to allow for up to eight treatment groups and allow treatment costs to vary between patients depending on patients’ compliance with atorvastatin and omega-3.[a] We therefore manually calculated the lifetime therapy costs in the extrapolated period by multiplying the life expectancy predicted by the model for each patient in each bootstrap by the annual cost of statins and omega-3 estimated for that patient based on the proportion of drug doses that they took during the 16-week trial. Total complication costs from the model were added to lifetime therapy costs to give the total cost during extrapolated period.

Similarly, total costs and QALYs for each patient were calculated by adding within-trial QALYs to the QALYs accrued over the extrapolated period for each patient extrapolated using each set of bootstrapped model parameters.

We combined parameter uncertainty around the UKPDS-OM2 risk equation parameters with sampling uncertainty around the risk factors and treatment effects within the AFORRD sample by bootstrapping from the AFORRD sample as well as extrapolating data using different sets of bootstrapped UKPDS-OM2 risk equation parameters (Method 5 within the recent methodological paper[20]). This method was used in preference to the other methods for combining sampling and parameter uncertainty since it facilitated adjustment for covariates and estimation of cost-effectiveness acceptability curves. We drew 10 bootstrap samples from AFORRD for each of the

[a] UKPDS-OM version 2.1 now has the option to model up to 25 different treatment groups, although that version of the software was not available at the time when the analysis for this paper were conducted.

1000 sets of UKPDS-OM2 parameters, giving a total of 10,000 different bootstrap samples. We extrapolated outcomes once for each of the 732 patients, before drawing bootstrap samples from AFORRD. Bootstrapping was stratified by patients' allocation to one of the eight combinations of three study interventions, such that each of the eight groups in the 2x2x2 design were bootstrapped separately. The Stata code used to run bootstrapping is shown in Appendix 2.

Linear regression was conducted on each bootstrap to control for covariates and (where appropriate) interactions between factors. Controlling for pre-randomisation risk factors avoids the bias that could otherwise result from imbalance between randomised groups.[20] With the exception of the sensitivity analyses controlling for fewer covariates, all analyses controlled for pre-randomisation values of all UKPDS-OM2 risk factors that vary between patients: non-white ethnicity, gender, age, duration of diabetes, BMI, history of atrial fibrillation, smoking, HDL-C, LDL-C, blood pressure and HbA1c. We did not control for cardiovascular events, albuminuria or peripheral vascular disease, since we assumed that none of the AFORRD participants had a history of these events. Similarly, we did not control for heart rate, white blood cell count, haemoglobin and glomerular filtration rate since these data were not collected in AFORRD and were set to the same values for all patients.

All of the three main analyses were conducted on the same set of bootstraps, as were the analyses used to generate Table 4 in Appendix 3 and most sensitivity analyses (Tables 6-8; Appendix 3). Each of the 10,000 bootstraps was analysed using three linear regression models to estimate results for the three main analyses:

- Analysis 1 (2x2) controlled for three treatment indicators: statin; omega-3; and statin*omega-3 interaction.
- Analysis 2 (2x2x2) included seven treatment indicators: statin; omega-3; action-planning; statin*omega-3 interaction; statin*action-planning interaction; omega-3*action-planning interaction; and the three-way statin*omega-3*action-planning interaction.
- Analysis 3 (assuming independence) included three treatment indicators: statin, omega-3 and action-planning.

We did not consider any interactions between treatment and risk factors to avoid making presentation and interpretation of results unnecessarily complicated and because considering interactions between 11 risk factors and up to seven treatment indicators would have introduced up to 77 additional interaction terms, which would have been too many to reliably estimate coefficients

in this trial of 732 patients. Furthermore, although such treatment-risk factor interactions could have given insights into heterogeneity (if the sample size had been large enough), allowing for such interactions is not necessary to avoid the bias resulting from baseline imbalance.

Before estimating linear regression, all continuous risk factors were "centred" by subtracting the overall mean (across all treatment groups) from each value to make it easier to generate meaningful predictions of outcomes for each treatment group. Binary risk factors (gender, ethnicity, smoking, and atrial fibrillation) were defined such that 0 represented the most common value for each of these risk factors.

Once bootstrapping was completed, we predicted outcomes for each treatment arm in each bootstrap based on the linear regression coefficients for the constant, treatment dummies and any between-treatment interactions. For example, the mean costs in bootstrap 1 for the group allocated to atorvastatin plus omega-3 equalled the constant term, plus the coefficient for atorvastatin, plus the coefficient for omega-3, plus (in Analyses 1 and 2) the atorvastatin*omega-3 interaction term. For Analysis 3 (assuming independence), we estimated the mean outcomes with(out) treatment X as the average of the predicted outcomes for the four groups (not) receiving that treatment.

Because of the centring, the mean values presented in Tables 1-3 in the main manuscript and Appendix 3, Table 4 represent the outcomes for white female non-smokers without atrial fibrillation who have the mean values for age, duration of diabetes, BMI, HDL-C, LDL-C, blood pressure and HbA1c. The absolute mean for each group would vary between risk factors, although because we used linear models with no treatment-risk factor interactions, the differences between groups and interactions between treatments were assumed to be unaffected by patients' baseline characteristics.

The mean values presented in this paper represent the mean across all 1000 bootstraps (rather than outcomes generated using the point estimates for each risk equation parameter) in order to present the expected net monetary benefit (NMB), allowing for non-linear relationships between risk equation parameters and lifetime costs or QALYs.

Expected NMB for each treatment was primarily calculated at a £20,000/QALY ceiling ratio[22] and the treatment with the highest NMB at a £20,000/QALY ceiling ratio was considered to represent best value for money. For Analyses 1 and 2, we calculated the percentage of all 10,000 bootstraps in

which each of the four or eight treatment combinations had highest NMB at different ceiling ratios and presented the data as cost-effectiveness acceptability curves. For Analysis 3, cost-effectiveness acceptability curves were calculated as the proportion of bootstraps in which the incremental NMB for treatment versus no treatment was positive. Methods for value of information calculations are presented in Appendix 3.

For costs and QALYs, the regression coefficients on each treatment dummy and their SEs are presented as the "simple effects" of each treatment in Tables 1 and 2 and as the "main effects" in Table 3. Once we had calculated the NMB for treatment arm x in each bootstrap (y_x), we manually calculated differences between groups for each bootstrap and estimated two (I_{AB}) and three-way (I_{ABC}) interactions for each bootstrap as:

$$I_{AB} = y_0 - y_a - y_b + y_{ab}$$
$$I_{ABC} = y_a + y_b + y_c + y_{ab} \ - y_0 - y_{ab} - y_{ac} - y_{bc} \quad (1)$$

SEs were calculated by taking the standard deviation across all 10,000 bootstraps and 95% CI were calculated from the SEs assuming a normal distribution. The statistical significance of treatment effects and interaction terms for costs and QALYs was based on the bootstrap p-value for the regression coefficient, and the statistical significance of NMB was based on bootstrap p-values for the differences and interaction terms estimated using the methods described in the paragraph above. When the difference or interaction was positive (negative), two-sided p-values were estimated as double the proportion of all 10,000 bootstraps that had differences or interactions above (below) 0.

References for Appendix 1

1. Palmer AJ, Si L, Tew M, et al. Computer Modeling of Diabetes and Its Transparency: A Report on the Eighth Mount Hood Challenge. *Value Health* 2018;21:724-31.

2. National Institute for Health and Care Excellence. British National Formulary. 2019 https://bnf.nice.org.uk/. Accessed 12 June 2019.

3. Curtis LA, Burns A. Unit Costs of Health and Social Care 2018. 2018 https://doi.org/10.22024/UniKent/01.02.70995. Accessed 4 March 2019.

4. Royal Mail. 1st and 2nd Class Stamps. 2019 https://shop.royalmail.com/postage-and-packaging/first-and-second-class-stamps. Accessed 12 June 2019.

5. Curtis LA, Burns A. Unit Costs of Health and Social Care 2015. 2015 https://www.pssru.ac.uk/project-pages/unit-costs/unit-costs-2015/. Accessed 12 July 2016.

6. Curtis L. Unit Costs of Health and Social Care 2010. 2010 http://www.pssru.ac.uk/pdf/uc/uc2010/uc2010.pdf. Accessed 9 December 2010.

7. Department of Health and Social Care. National Schedule of Reference Costs - Year 2014-15 - NHS trusts and NHS foundation trusts: the main schedule. 2015 https://www.gov.uk/government/publications/nhs-reference-costs-2014-to-2015. Accessed 15 August 2016.

8. Department Of Health NHS Commissioning Board. General Ophthalmic Services – increases to NHS Sight Test Fee, continuing education and training payment and pre-registration supervisors grant. 2015 https://www.gov.uk/government/uploads/system/uploads/attachment_data/file/453364/Sight_Test_Fee_Letter_acc.pdf Accessed 19 June 2019.

9. Farmer AJ, Oke J, Hardeman W, et al. The effect of a brief action planning intervention on adherence to double-blind study medication, compared to a standard trial protocol, in the Atorvastatin in Factorial with Omega EE90 Risk Reduction in Diabetes (AFORRD) clinical trial: A cluster randomised sub-study. *Diabetes Res Clin Pract* 2016;120:56-64.

10. Health & Social Care Information Centre. Prescription Cost Analysis, England - 2015: Data csv (PCA inc Dental). 2016 http://www.hscic.gov.uk/catalogue/PUB20200/pres-cost-anal-eng-2015-data.csv. Accessed 6 June 2016.

11. Dolan P. Modeling valuations for EuroQol health states. *Med Care* 1997;35:1095-108.

12. Holman RR, Paul S, Farmer A, et al. Atorvastatin in Factorial with Omega-3 EE90 Risk Reduction in Diabetes (AFORRD): a randomised controlled trial. *Diabetologia* 2009;52:50-9.

13. Stevens RJ, Kothari V, Adler AI, et al. The UKPDS risk engine: a model for the risk of coronary heart disease in Type II diabetes (UKPDS 56). *Clinical science* 2001;101:671-9.

14. UKPDS Risk Engine version 2.0. http://www.dtu.ox.ac.uk/riskengine/index.php. Accessed 6 October 2008.

15. Hayes AJ, Leal J, Gray AM, et al. UKPDS outcomes model 2: a new version of a model to simulate lifetime health outcomes of patients with type 2 diabetes mellitus using data from the 30 year United Kingdom Prospective Diabetes Study: UKPDS 82. *Diabetologia* 2013;56:1925-33.

16. Alva ML, Gray A, Mihaylova B, et al. The impact of diabetes-related complications on healthcare costs: new results from the UKPDS (UKPDS 84). *Diabet Med* 2015;32:459-66.

17. National Institute for Health and Care Excellence. Guide to the methods of technology appraisal 2013. 2013 https://www.nice.org.uk/process/pmg9/chapter/foreword. Accessed 16 April 2020.

18. Alva M, Gray A, Mihaylova B, et al. The effect of diabetes complications on health-related quality of life: the importance of longitudinal data to address patient heterogeneity. *Health Econ* 2014;23:487-500.

19. University of Oxford Diabetes Trials Unit (DTU) and Health Economics Research Centre (HERC). UKPDS Outcomes Model User Manual: Version 2.0. 2015 https://www.dtu.ox.ac.uk/outcomesmodel/OM2Manual.pdf. Accessed 16 November 2016.

20. Dakin H, Leal J, Briggs A, et al. Handling uncertainty when using patient-level simulation models to extrapolate clinical trial data. *Med Decis Making (in press)* 2020.

21. O'Hagan A, Stevenson M, Madan J. Monte Carlo probabilistic sensitivity analysis for patient level simulation models: efficient estimation of mean and variance using ANOVA. *Health Econ* 2007;16:1009-23.

22. National Institute for Health and Clinical Excellence. Social value judgements: Principles for the development of NICE guidance. Second Edition. 2008 http://www.nice.org.uk/media/C18/30/SVJ2PUBLICATION2008.pdf. Accessed 31st May 2012.
