# Supplementary material for: Economic Evaluation of Factorial Trials: Cost-Utility Analysis of the Atorvastatin in Factorial With Omega EE90 Risk Reduction in Diabetes 2 × 2 × 2 Factorial Trial of Atorvastatin, Omega-3 Fish Oil, and Action Planning
Source: Value Health. 2020 Oct;23(10):1340–8. doi: 10.1016/j.jval.2020.05.018 (PMC7537832; doi:10.1016/j.jval.2020.05.018)
Supplement: Appendix 3 [file mmc5.pdf]

Appendix 3: Additional results

Table 3. Within-trial resource use and costs

	Ambulatory consultations		Hospitalisations		Concomitant medications		Study treatment	Total within-trial cost
	**No. visits**	**Cost**	**No. days**	**Cost**	**No. days**	**Cost**	**Cost**	
Unadjusted means and standard deviations (SD) for each group								
No treatment (SD)	4.28 (2.59)	£146.60 (£93.26)	0.09 (0.71)	£44.21 (£333.15)	563.02 (343.44)	£138.52 (£118.73)	£0.00 (£0.00)	£329.33 (£389.72)
Atorvastatin (SD)	4.10 (2.60)	£144.84 (£111.56)	0.04 (0.31)	£59.38 (£478.01)	492.13 (272.87)	£119.08 (£95.78)	£2.89 (£0.54)	£326.18 (£519.03)
Omega-3 (SD)	4.96 (4.61)	£169.40 (£161.02)	0.14 (1.18)	£76.21 (£492.43)	507.57 (313.03)	£121.70 (£122.94)	£90.58 (£28.15)	£457.90 (£541.84)
Atorvastatin+omega-3 (SD)	4.62 (3.21)	£167.98 (£123.36)	0.25 (1.87)	£116.33 (£794.66)	552.23 (403.65)	£174.25 (£437.72)	£97.23 (£23.41)	£555.80 (£924.48)
Action-planning (SD)	3.27 (2.28)	£113.35 (£82.92)	0.08 (0.60)	£152.35 (£922.16)	499.60 (298.58)	£113.12 (£90.54)	£4.32 (£0.00)	£383.14 (£948.82)
Atorvastatin+action-planning (SD)	3.98 (3.41)	£120.59 (£126.32)	0.00 (0.00)	£0.00 (£0.00)	515.18 (344.23)	£127.16 (£130.0)	£7.25 (£0.48)	£255.01 (£199.95)
Omega-3+action-planning (SD)	4.56 (3.90)	£143.69 (£121.81)	0.04 (0.34)	£21.36 (£186.18)	440.28 (315.57)	£113.91 (£103.34)	£100.92 (£26.84)	£379.88 (£248.59)
Atorvastatin+omega-3+action-planning (SD)	4.76 (3.65)	£140.39 (£116.50)	0.01 (0.11)	£21.00 (£185.43)	517.12 (302.92)	£121.27 (£100.08)	£103.71 (£24.71)	£386.36 (£266.61)
Incremental effects and interactions and standard errors (SE), adjusted† for all baseline UKPDS-OM2 risk factors using linear regression								
Atorvastatin vs no treatment (SE)	0.02 (0.36)	£4.85 (£14.18)	-0.05 (0.08)	£14.92 (£57.54)	-42.08 (43.11)	-£2.06 (£18.62)	£3.06 (£0.65)*	£20.77 (£64.86)
Omega-3 vs no treatment (SE)	0.87 (0.54)	£30.21 (£19.23)	0.05 (0.14)	£29.37 (£56.23)	-29.99 (44.76)	-£3.29 (£17.98)	£90.95 (£2.72)*	£147.23 (£64.46)*
Action-planning vs no treatment SE)	-0.77 (0.39)*	-£23.50 (£14.49)	0.00 (0.09)	£118.55 (£110.82)	-19.06 (47.38)	-£0.76 (£19.77)	£4.02 (£0.83)*	£98.31 (£116.16)
Inter-action: Atorvastatin*omega-3 (SE)	-0.36 (0.67)	-£5.03 (£24.36)	0.17 (0.22)	£28.69 (£101.04)	92.07 (63.82)	£55.32 (£37.23)	£3.14 (£3.51)	£82.13 (£114.20)
Atorvastatin*action-planning (SE)	0.64 (0.60)	-£0.67 (£22.50)	-0.02 (0.10)	-£166.87 (£124.17)	32.96 (64.73)	£10.89 (£25.66)	-£0.26 (£0.87)	-£156.91 (£132.22)
Omega-3*action-planning (SE)	0.51 (0.71)	£4.65 (£24.28)	-0.11 (0.16)	-£172.05 (£123.77)	-24.11 (64.02)	£1.81 (£23.96)	£5.22 (£3.97)	-£160.38 (£131.07)
Atorvastatin*omega-3*action-planning (SE)	-0.25 (0.99)	-£8.68 (£34.97)	-0.10 (0.25)	£134.44 (£156.73)	-22.82 (92.25)	-£57.25 (£43.93)	-£2.78 (£5.49)	£65.72 (£171.03)

* p<0.05

† The incremental effects and interactions shown in the lower half of the table were estimated using a linear regression model controlling for all three randomised treatment allocations, all interactions and pre-randomisation values of the following UKPDS-OM2 risk factors: non-white ethnicity, gender, age, duration of diabetes, BMI, history of atrial fibrillation, smoking, HDL-C, LDL-C, blood pressure and HbA1c. Consequently, the adjusted differences between groups differ slightly from the absolute differences between the unadjusted group means shown in the top half of the table.

Table 4. Mean costs and standard errors for disaggregated outcomes during the extrapolated period. Estimated using the same methods and the same set of bootstraps applied to total costs and total QALYs.

		Therapy costs	Complication costs	Life years
No treatment		£108 (£78)	£28,995 (£582)	12.687 (0.157)
Atorvastatin		£133 (£67)	£29,026 (£561)	13.075 (0.163)
Omega-3		£3,720 (£136)	£29,023 (£577)	12.709 (0.158)
Atorvastatin+omega-3		-£39 (£83)	£29,645 (£601)	12.732 (0.161)
Action-planning		£4,075 (£124)	£28,947 (£582)	13.034 (0.167)
Atorvastatin+action-planning		£153 (£81)	£28,864 (£584)	12.972 (0.168)
Omega-3+action-planning		£4,266 (£153)	£29,366 (£596)	12.732 (0.159)
Atorvastatin+omega-3+ action-planning		£4,272 (£150)	£29,268 (£573)	13.095 (0.167)
Atorvastatin simple effect		£25 (£89)	£31 (£285)	0.388 (0.071)*
Omega-3 simple effect		£3,612 (£143)*	£29 (£297)	0.022 (0.061)
Action-planning simple effect		-£147 (£100)	£651 (£306)*	0.045 (0.064)
Interaction	Atorvastatin by omega-3	£330 (£184)	-£107 (£413)	-0.063 (0.086)
	Atorvastatin by action-planning	£167 (£134)	-£813 (£425)	-0.148 (0.093)
	Omega-3 by action-planning	£693 (£208)*	-£308 (£444)	-0.022 (0.091)
	Atorvastatin by omega-3 by action-planning	-£516 (£287)	£791 (£601)	0.186 (0.124)

Values represent the mean (standard error) for each group for white female non-smokers without atrial fibrillation who have the mean values for age, duration of diabetes, body mass index, high-density lipoprotein, low-density lipoprotein, blood pressure and glycated haemoglobin (HbA1c).

Fig. 2. Point estimates from (A) Analysis 1 (2x2) and (B) Analysis 2 (2x2x2).

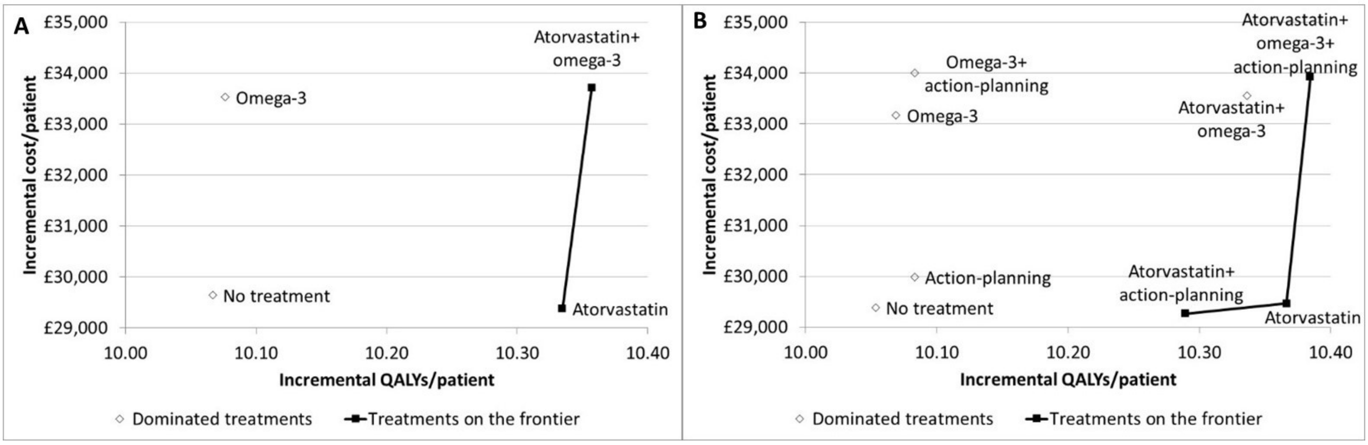

The frontier (shown with the black lines) joins the treatments that are best value for money (have highest net monetary benefit) at different ceiling ratios. QALYs, quality-adjusted life-years.

Fig. 3. Cost-effectiveness acceptability curves for Analysis 3 (assuming independence) for (A) omega-3 versus no omega-3 and (C) action-planning versus no action-planning base case.

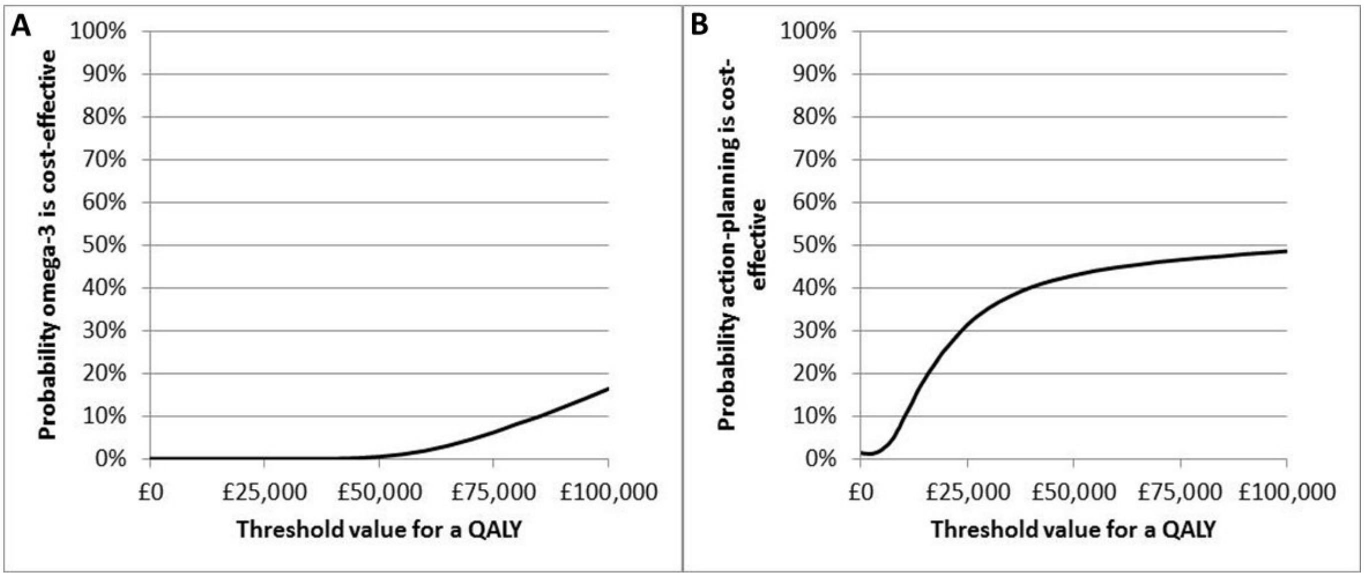

Expected value of perfect information (EVPI)

The EVPI per patient was estimated by identifying the highest NMB in each bootstrap across the four (or eight, or two) treatment arms, averaging this figure over the 10,000 bootstraps and subtracting the mean NMB for the treatment arm with highest expected NMB.

Fig. 4. Expected value of perfect information per patient for Analysis 1, 2x2; Analysis 2 2x2x2; and each comparison in Analysis 3

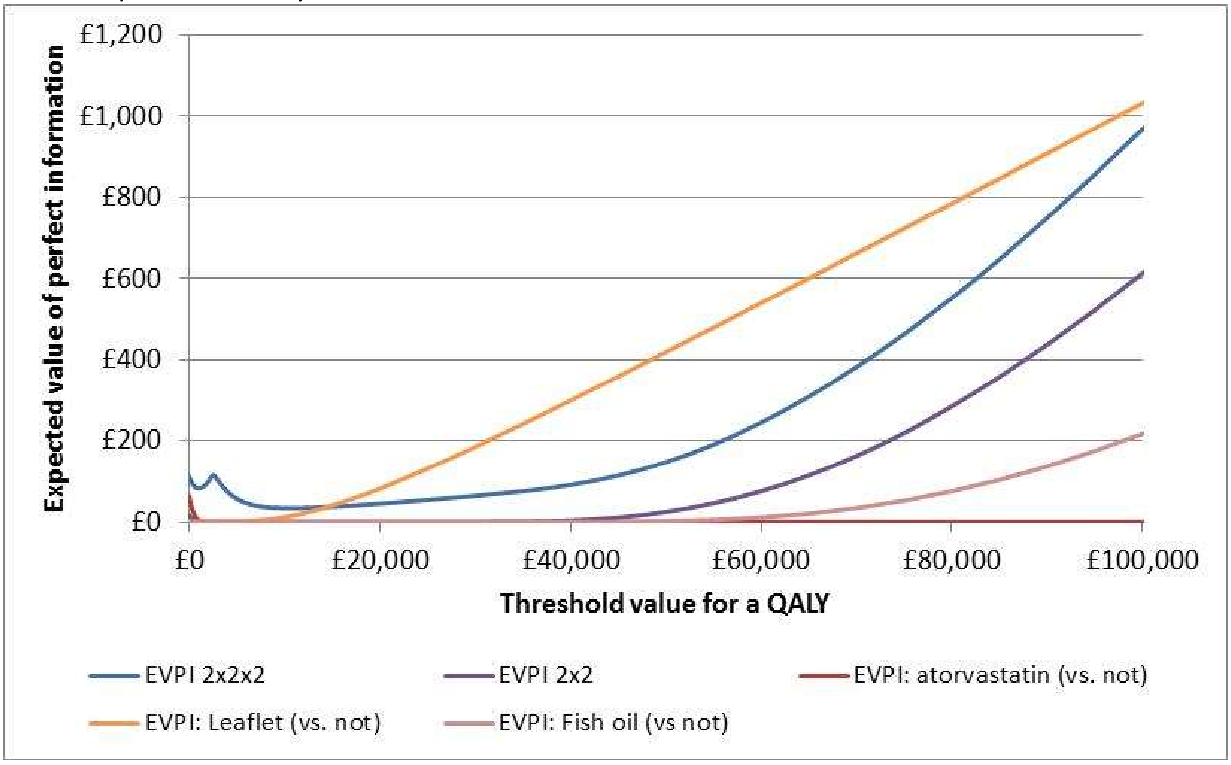

We extrapolated the EVPI per patient at a £20,000/QALY ceiling ratio to the UK population based on the epidemiological data shown in Table 5. This analysis assumed that further information on the efficacy and cost-effectiveness of statins, omega-3 fish oil and/or action-planning leaflets would be valid for 10 years and applied to all adults with type 2 diabetes except those who are contraindicated to statins. We also assumed that the interventions would be given to all prevalent diabetes patients in Year 1 and all incident cases as they were diagnosed.

Conclusions in the text focus on the EVPI for the 2x2x2 analysis, since Analysis 3 (assuming independence) is prone to bias due to ignoring interactions (which is of particular relevance for the action-planning leaflet, which is only expected to be effective for patients receiving atorvastatin). Since the results of Analyses 1 and 3 demonstrate that there is no uncertainty around whether atorvastatin and omega-3 fish oil are cost-effective at a £20,000/QALY ceiling ratio, it is reasonable to assume that any further research should be directed at the action-planning intervention.

Table 5. Calculation of population expected value of perfect information (EVPI) at a £20,000/QALY ceiling ratio

Number of prevalent diabetes cases in UK (2009)	2,600,000	Diabetes UK[1]
Annual number of incident diabetes cases (2008)	145,000	Diabetes UK[1]
Number of prevalent cases of diabetes in children	26,400	Diabetes UK[1]
Proportion of adult diabetes cases that are type 2	90%	Diabetes UK[1]
Estimated proportion of the annual incident cases that arise in children rather than adults	1%	Assumed to be the same proportion as for prevalent cases
Proportion of patients contraindicated the statins	3%	SIGN costing template[2]
Discount rate	3.5%	NICE reference case[3]
Total number of prevalent type 2 diabetes cases in adults	2,246,753	Calculated from above figures
Annual number of incident cases of type 2 diabetes in adults	125,300	Calculated from above figures
Total cases over 10 years, discounted at 3.5% per annum	3,325,293	Calculated from above figures
	**Per patient**	**For UK over 10 years**
EVPI: 2x2 analysis	£0	£0
EVPI: 2x2x2 analysis	£45	£149,435,281
EVPI: assuming independence - atorvastatin vs. no atorvastatin	£0	£0
EVPI: assuming independence - omega-3 vs. omega-3	£0	£0
EVPI: assuming independence - action-planning vs. no action-planning	£82	£272,599,746

Opportunity cost of ignoring interactions

We followed the methods of Dakin and Gray 2017[4] to calculate the opportunity cost of ignoring interactions under current information (Fig. 5). This is a measure of the value of taking account of interactions when conducting economic evaluations on factorial trials or (conversely) the opportunity cost of ignoring interactions. This was only calculated for Analysis 3 (assuming independence), since Analysis 1 (2x2) only considered two of the three interventions, making it difficult to interpret this measure meaningfully. The opportunity cost was calculated by taking the difference between the expected NMB for the treatment combination that would be adopted in an analysis taking account of all interactions and the expected NMB for the treatment combination that will be adopted in Analysis 3. When calculating this measure, all NMBs were based on the results of Analysis 2 (2x2x2) for consistency; we therefore took the highest NMB across the eight treatment combinations considered in Analysis 2 and subtracted the Analysis 2 estimate of the combination of treatments with positive INB in Analysis 3.

Fig. 5. Opportunity cost of ignoring interactions: Analysis 3 (assuming independence) compared with Analysis 2 (2x2x2)

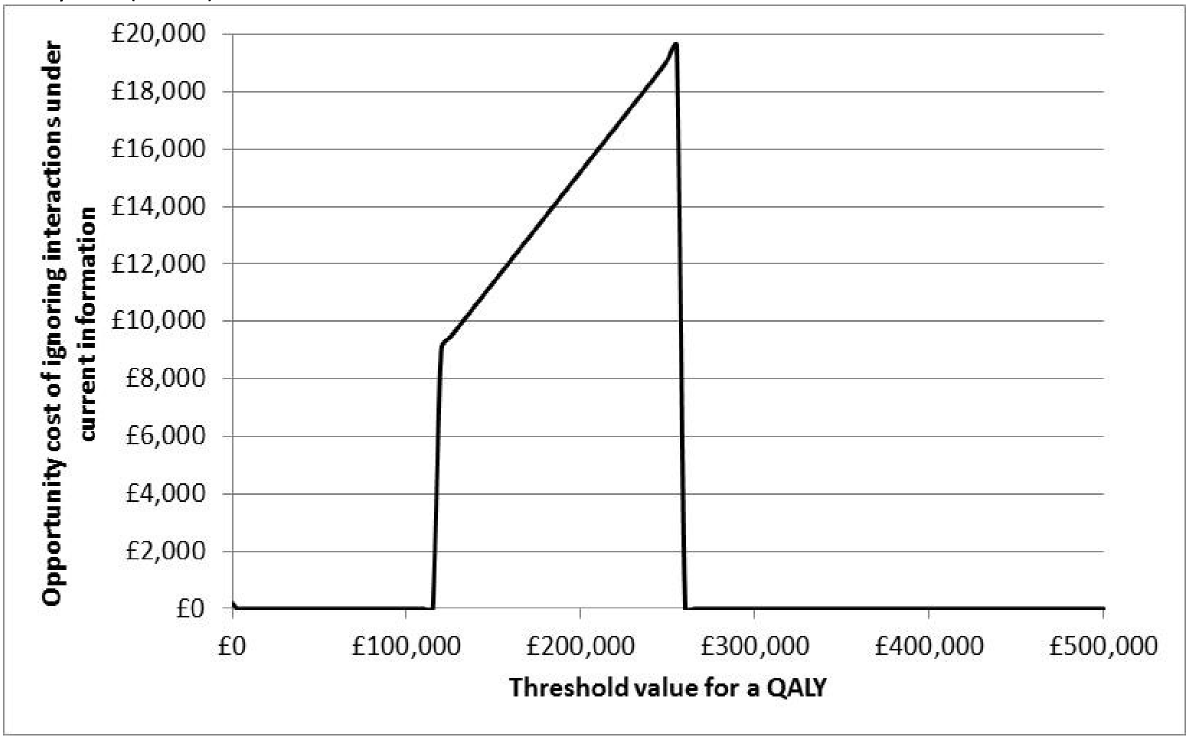

Methods and results of sensitivity analyses

The following sensitivity analyses were conducted on the same run of UKPDS-OM2 and the same set of bootstraps from AFORRD that were used for the base case analysis. This eliminates chance

differences between sensitivity analyses. Unless otherwise specified, the methods used in each sensitivity analysis were the same as the base case analysis. Results are presented in Tables 6-8.

- Ignoring sampling uncertainty: linear regression for each set of UKPDS-OM2 parameters was conducted on the original AFORRD sample, without bootstrapping the AFORRD sample. This analysis controlled for all baseline UKPDS-OM2 parameters and allocation to all three trial interventions and is therefore not directly comparable to the first analysis presented within the recent methodological paper.[5] When sampling uncertainty was excluded without controlling for baseline UKPDS-OM2 parameters, the incremental NMB for atorvastatin versus no atorvastatin was £6,058 (SE: £740), vs. £5,526 (SE: £919) in the base case analysis and £6048 (SE: £4,413) in an additional sensitivity analysis including sampling uncertainty without controlling for covariates. The SE around this incremental NMB in the analysis excluding sampling uncertainty without controlling for baseline UKPDS-OM2 parameters was 80.6% (740/919) of the size of the SE around the incremental NMB in the base case analysis; this is similar to the ratio of SEs around life years gained in Analysis 1 (0.06921) and Analysis 4b (0.10270) in the methodological paper (67%).
- No allowance for covariates: the analysis controlled for no variables other than treatment indicators and interaction terms (cf. the base case analysis which controlled for baseline UKPDS-OM risk factors).
- Excluding within-trial costs and QALYs: Regression analyses were run solely on the estimated costs and QALYs in the extrapolated period, without first adding on the within-trial costs and QALYs.
- 2015/16 prices: Regression models were rerun using 2015/16 prices for study interventions, with no adjustment for inflation. Atorvastatin cost £0.0443/day [53% more than base case], omega-3 EE90 cost £1.0168/day [0.03% less than base case] and action-planning had a one-off cost of £4.5333 [4.9% more than base case].
- Basing therapy costs on 100% compliance: The costs of atorvastatin and omega-3 during the trial and during the extrapolated period were calculated based on 100% compliance, rather than the compliance observed in the trial.
- Basing therapy costs on the average level of compliance to all patients: We calculated the mean level of compliance with atorvastatin and omega-3 across all 732 patients (85%) and used this level of compliance to calculate treatment costs for atorvastatin and omega-3 during the trial and during the extrapolated period, in place of the compliance values for individual patients. This analysis was implementing by adjusting the drug costs estimated in the analysis using full compliance to model 85% compliance rather than 100%.

- Changing the cost of the action-planning leaflet to 0%, 50% or 500% of the base case: This analysis was conducted simply by adding or subtracting the relevant value from the mean cost in each treatment arm, after all analyses had been run.

The UKPDS-OM2 extrapolation was rerun five times to generate the following sets of sensitivity analyses. To minimise computation time, the number of loops was set to 5,000 for each of the sensitivity analyses, although the same set of 1,000 bootstrapped UKPDS-OM2 risk equation parameters was used in all of the sensitivity analyses and in the base case analysis.

- No discounting
- Discounting at 3.5% for years 1-30 and 3% thereafter
- 10-year time horizon
- 30-year time horizon
- Allowing for risk factor progression built into 2.01b8: An updated version of the UKPDS-OM2 (version 2.01b8) was used in place of the 2.01b5 version used for the base case analysis (which assumed risk factors remain constant for a lifetime). In this sensitivity analysis, risk factors were assumed to change over time based on equations that predict how risk factors (e.g. HbA1c and smoking) change over time (Leal et al, personal communication of unpublished data).

Analysis 3 (assuming independence) was also re-analysed using separate regressions on each factor. In the base case analysis, we followed best practice[6] by controlling for all three factors simultaneously. This sensitivity analysis tested what the results would have been if we had instead conducted one regression analysis on atorvastatin versus no atorvastatin, one on omega-3 versus no omega-3 and one on action-planning versus no action-planning (mirroring the way that factorial trials ignoring interactions are often analysed). Each of the three analyses controlled for pre-randomisation risk factors. For practical purposes, we conducted this sensitivity analysis on a new set of bootstrapped samples drawn from AFORRD (not that used for the base case analysis), although all three regressions used in this sensitivity analysis used the same set of bootstraps. This analysis used the same set of extrapolated costs and QALYs used the base case analysis.

Table 6. Results of sensitivity analyses on the 2x2 analysis (Analysis 1)

	**No treatment**	**Atorvastatin**	**Omega-3**	**Atorvastatin+ omega-3**	**Probability atorvastatin alone is cost-effective**
	**Mean (standard error) net monetary benefit at £20,000/QALY ceiling ratio**				
Base case	***£171,691 (£2,116)***	***£177,312 (£2,245)***	***£167,996 (£2,082)***	***£173,433 (£2,254)***	***100%***
Ignoring sampling uncertainty	£171,680 (£2,043)	**£177,284 (£2,196)**	£168,024 (£1,997)	£173,421 (£2,197)	100%
No allowance for covariates	£172,028 (£4,747)	**£180,700 (£4,771)**	£173,133 (£4,683)	£176,750 (£4,596)	67%
10-year time horizon‡	£108,471 (£783)	**£110,236 (£760)**	£105,939 (£762)	£107,879 (£755)	100%
30-year time horizon‡	£165,969 (£1,931)	**£171,210 (£2,023)**	£162,439 (£1,892)	£167,615 (£2,043)	100%
Excluding within-trial costs and QALYs	£166,954 (£2,114)	**£172,523 (£2,239)**	£163,322 (£2,080)	£168,813 (£2,246)	100%
2015/16 prices	£172,561 (£2,125)	**£178,106 (£2,253)**	£168,864 (£2,091)	£174,239 (£2,262)	100%
No discounting‡	£242,441 (£4,331)	**£252,256 (£4,573)**	£237,058 (£4,314)	£246,306 (£4,549)	100%
Discounting at 3.5% for years 1-30 and 3% thereafter‡	£171,610 (£2,156)	**£177,297 (£2,294)**	£167,886 (£2,129)	£173,368 (£2,309)	100%
Allowing for risk factor progression built into 2.01b8‡	£155,167 (£1,637)	**£159,554 (£1,738)**	£151,621 (£1,612)	£155,635 (£1,740)	100%
Basing therapy costs on 100% compliance	£171,771 (£2,115)	**£177,385 (£2,244)**	£167,247 (£2,072)	£172,659 (£2,248)	100%
Estimating therapy costs by applying the average level of compliance (85%) to all patients	£171,759 (£2,115)	**£177,374 (£2,244)**	£167,359 (£2,074)	£172,775 (£2,248)	100%
	**Mean (SE) difference between each group and atorvastatin monotherapy**				-
Random effects by GP practice§	-£5,604 (£1,025)§	**£0 (baseline group)§**	-£9,260 (£1,041)§	-£3,863 (£689)§	100%

Values represent the mean (standard error) for each group for white female non-smokers without atrial fibrillation who have the mean values for age, duration of diabetes, body mass index, high-density lipoprotein, low-density lipoprotein, blood pressure and glycated haemoglobin (HbA1c). The treatment with highest NMB is shown in bold typeface.

‡ Based on a separate run of the UKPDS-OM2 and separate bootstrapping of AFORRD

§ Results for this row represent the mean (SE) difference between each group and atorvastatin monotherapy. Based on the same run of UKPDS-OM2, analysed using a mixed model with random effects on GP practice.

Table 7. Results of sensitivity analysis on the 2x2x2 analysis (Analysis 2)

	**No treatment**	**Atorvastatin**	**Omega-3**	**Action-planning**	**Atorvastatin+ omega-3**	**Atorvastatin+ action-planning**	**Omega-3+action-planning**	**Atorvastatin+ omega-3+ action-planning**	**Probability atorvastatin alone is cost-effective**
	**Mean (standard error) net monetary benefit at £20,000/QALY ceiling ratio**								
Base case analysis	***£171,676 (£2,163)***	***£177,866 (£2,279)***	***£168,205 (£2,152)***	***£171,674 (£2,201)***	***£171,674 (£2,201)***	***£176,524 (£2,337)***	***£167,662 (£2,140)***	***£173,769 (£2,277)***	***90%***
Ignoring sampling uncertainty	£171,663 (£2,040)	**£177,859 (£2,212)**	£168,217 (£1,994)	£171,666 (£2,054)	£173,161 (£2,201)	£176,464 (£2,176)	£167,711 (£2,011)	£173,751 (£2,196)	100%
No allowance for covariates	£164,095 (£6,138)	**£181,022 (£5,964)**	£162,648 (£5,995)	£183,438 (£6,829)	£174,062 (£6,029)	£180,257 (£7,175)	£187,894 (£6,839)	£180,542 (£6,238)	9%
10-year time horizon‡	£108,102 (£892)	**£110,428 (£810)**	£105,481 (£874)	£108,949 (£916)	£107,297 (£885)	£109,945 (£942)	£106,512 (£895)	£108,666 (£809)	70%
30-year time horizon‡	£165,397 (£1,993)	**£172,065 (£2,060)**	£162,113 (£1,970)	£166,680 (£2,131)	£166,766 (£2,165)	£169,972 (£2,267)	£162,767 (£2,039)	£168,730 (£2,095)	93%
Excluding within-trial costs and QALYs	£166,895 (£2,158)	**£173,099 (£2,274)**	£163,511 (£2,147)	£166,998 (£2,199)	£168,605 (£2,288)	£171,704 (£2,325)	£163,017 (£2,141)	£169,070 (£2,273)	92%
2015/16 prices	£172,537 (£2,172)	**£178,664 (£2,287)**	£169,069 (£2,161)	£172,556 (£2,210)	£173,974 (£2,312)	£177,312 (£2,345)	£168,535 (£2,149)	£174,575 (£2,285)	90%
No discounting‡	£243,937 (£4,550)	£252,246 (£4,644)	£239,187 (£4,589)	£240,438 (£4,776)	£247,846 (£4,748)	**£252,336 (£5,050)**	£234,274 (£4,638)	£244,209 (£4,743)	48%
Discounting at 3.5% for years 1-30 and 3% thereafter‡	£171,712 (£2,201)	**£177,779 (£2,340)**	£168,216 (£2,201)	£171,440 (£2,252)	£173,233 (£2,353)	£176,616 (£2,367)	£167,400 (£2,192)	£173,530 (£2,343)	87%
Allowing for risk factor progression built into 2.01b8‡	£155,296 (£1,688)	**£159,947 (£1,763)**	£152,083 (£1,664)	£154,964 (£1,698)	£155,574 (£1,785)	£159,001 (£1,812)	£150,963 (£1,679)	£155,701 (£1,769)	88%
Basing therapy costs on 100% compliance	£171,709 (£2,163)	**£177,921 (£2,278)**	£167,361 (£2,138)	£171,818 (£2,201)	£172,349 (£2,298)	£176,619 (£2,336)	£167,040 (£2,132)	£173,057 (£2,274)	89%
Estimating therapy costs by applying the average level of compliance (85%) to all patients	£171,704 (£2,163)	**£177,913 (£2,278)**	£167,488 (£2,139)	£171,797 (£2,201)	£172,472 (£2,299)	£176,605 (£2,336)	£167,133 (£2,133)	£173,164 (£2,274)	89%

	No treatment	Atorvastatin	Omega-3	Action-planning	Atorvastatin+ omega-3	Atorvastatin+ action-planning	Omega-3+action-planning	Atorvastatin+ omega-3+ action-planning	Probability atorvastatin alone is cost-effective
	**Mean (standard error) net monetary benefit at £20,000/QALY ceiling ratio**								
Base case analysis	***£171,676 (£2,163)***	***£177,866 (£2,279)***	***£168,205 (£2,152)***	***£171,674 (£2,201)***	***£171,674 (£2,201)***	***£176,524 (£2,337)***	***£167,662 (£2,140)***	***£173,769 (£2,277)***	***90%***
Excluding the cost of the action-planning leaflet	£171,676 (£2,163)	**£177,866 (£2,279)**	£168,205 (£2,152)	£171,678 (£2,201)	£173,169 (£2,304)	£176,528 (£2,337)	£167,666 (£2,140)	£173,773 (£2,277)	90%
Decreasing the cost of the action-planning leaflet to 50% of the base case (£2.16/patient)	£171,676 (£2,163)	**£177,866 (£2,279)**	£168,205 (£2,152)	£171,676 (£2,201)	£173,169 (£2,304)	£176,526 (£2,337)	£167,664 (£2,140)	£173,771 (£2,277)	90%
Increasing the cost of the action-planning leaflet to 500% of the base case (£21.60/patient)	£171,676 (£2,163)	**£177,866 (£2,279)**	£168,205 (£2,152)	£171,657 (£2,201)	£173,169 (£2,304)	£176,506 (£2,337)	£167,644 (£2,140)	£173,752 (£2,277)	90%
	**Mean (SE) difference between each group and atorvastatin monotherapy**								**-**
Random effects by GP practice§	-£6,196 (£1,174)§	**£0 (baseline group)**§	-£9,642 (£1,230)§	-£6,193 (£1,302)§	-£4,698 (£901)§	-£1,395 (£985)§	-£10,148 (£1,252)§	-£4,108 (£984)§	92%

Values represent the mean (standard error) for each group for white female non-smokers without atrial fibrillation who have the mean values for age, duration of diabetes, body mass index, high-density lipoprotein, low-density lipoprotein, blood pressure and glycated haemoglobin (HbA1c). The treatment with highest NMB is shown in bold typeface.

‡ Based on a separate run of the UKPDS-OM2 and separate bootstrapping of AFORRD.

§ Results for this row represent the mean (SE) difference between each group and atorvastatin monotherapy. Based on the same run of UKPDS-OM2, analysed using a mixed model with random effects on GP practice.

Table 8. Results of sensitivity analysis on Analysis 3 (assuming independence)

	**Mean incremental net monetary benefit at £20,000/QALY ceiling ratio (standard error; probability that intervention is cost-effectiveness with comparator)**		
	**Atorvastatin versus no atorvastatin**	**Omega-3 versus no omega-3**	**Action-planning versus no action-planning**
Base case analysis	£5,526 (£919; 100%)	-£3,788 (£495; 0%)	-£323 (£512); 26%)
Ignoring sampling uncertainty	£5,497 (£776; 100%)	-£3,760 (£97; 0%)	-£329 (£114; 0%)
No allowance for covariates	£6,048 (£4,413; 91%)	-£1,452 (£4,327; 37%)	£12,484 (£4,381; 100%)
10-year time horizon‡	£1,858 (£481; 100%)	-£2,444 (£431; 0%)	£685 (£433; 95%)
30-year time horizon‡	£5,211 (£959; 100%)	-£3,564 (£658; 0%)	£437 (£687; 74%)
Excluding within-trial costs and QALYs	£5,527 (£912; 100%)	-£3,671 (£485; 0%)	-£335 (£505; 25%)
2015/16 prices	£5,457 (£918; 100%)	-£3,783 (£495; 0%)	-£318 (£512; 26%)
No discounting‡	£9,510 (£2,113; 100%)	-£5,664 (£1,568; 0%)	-£2,958 (£1,569; 3%)
Discounting at 3.5% for years 1-30 and 3% thereafter‡	£5,580 (£930; 100%)	-£3,828 (£515; 0%)	-£487 (£518; 17%)
Allowing for risk factor progression built into 2.01b8‡	£4,195 (£655; 100%)	-£3,734 (£412; 0%)	-£564 (£425; 9%)
Basing therapy costs on 100% compliance	£5,510 (£917; 100%)	-£4,626 (£495; 0%)	-£204 (£512; 35%)
Estimating therapy costs by applying the average level of compliance (85%) to all patients	£5,512 (£917; 100%)	-£4,501 (£494; 0%)	-£222 (£512; 33%)
Conducting three separate regressions rather than controlling for all three treatments in one regression†	£5,507 (£911; 100%)	-£3,776 (£497; 0%)	-£357 (£521; 24%)
Random effects by GP practice§	£5,497 (£914; 100%)	-£3,760 (£494; 0%)	-£329 (£511; 26%)

Values represent the mean (standard error) for each group for white female non-smokers without atrial fibrillation who have the mean values for age, duration of diabetes, body mass index, high-density lipoprotein, low-density lipoprotein, blood pressure and glycated haemoglobin (HbA1c).
† Based on the same run of UKPDS, but a separate set of bootstraps drawn from AFORRD.
‡ Based on a separate run of the UKPDS-OM2 and separate bootstrapping of AFORRD.
§ Based on the same run of UKPDS, analysed with a mixed model.

We also conducted a sensitivity analysis using random effects by GP practice. Since a two-stage bootstrap would have been infeasible with the small clusters used in AFORRD,[a] the analysis was conducted using mixed models with random effects on GP practice. The Stata prefix `mi estimate` was used to run regression models on each of the 1000 bootstraps and combine the results using Rubin's rule. This approach combines parameter uncertainty around the UKPDS-OM2 risk equation parameters with sampling uncertainty around the risk factors and treatment effects within the AFORRD sample.[5] In this context, the 1000 estimates of lifetime costs and QALYs generated for each patient within the UKPDS-OM2 using different bootstrapped risk equation parameters were treated as being analogous to 1000 imputed datasets.[5]

Mixed models (estimated using Stata's `mixed` command) were used to allow for the clustering of patients by GP practice. The model included random constants for each GP practice (ρ_g) to allow for clustering of patients by GP practice and allow for the possibility that centres may vary in how the cluster-randomised interventions (e.g. action-planning) are administered.[7,8] We did not evaluate random slopes (i.e. practice-specific treatment effects) in this analysis since these models were unlikely to converge as our analyses included up to 8 treatment groups and some GP practices recruited only 2 patients. Practice effects were ignored in the base case analysis as such variations are unlikely within the first 16 weeks of AFORRD, since all patients were posted the same action-planning leaflet and no reinforcement was received until after 16 weeks.

The model for Analysis 3 (assuming independence) therefore predicted NMB as:

$$\theta_{g,b} = \beta_0 + \beta_A A + \beta_F F + \beta_L L + \sum_{c=1}^{C} \beta_c C + \rho_g + \varepsilon_{g,b} \qquad (2)$$

Where $\theta_{g,b}$ represents the lifetime NMB for bootstrap *b* of patient *i* who is in centre *g*. C represents the list of covariates (e.g. age, gender). A indicates randomised allocation to atorvastatin, F indicates randomised allocation to omega-3 fish oil and L indicates randomised allocation to the action-planning leaflet.

[a] A two-stage bootstrap on the 2x2x2 analysis would have involved the following procedure. We would first sample, with replacement, 30 bootstrap samples of 30 practices from the 30 GP practices randomised to action planning, and repeat this for the 28 GP practices randomised to no action planning. (One of the 59 practices did not recruit any patients with sufficient complete data to include in our analysis.) We would then need to sample, with replacement, $n_{k,j}$ patients from each of the four combinations of individually-randomised allocations (no treatment, atorvastatin, omega-3 and atorvastatin+omega-3), where $n_{k,j}$ equals the number of patients recruited to combination *k* in practice *j*. This second level of bootstrapping would not be feasible for any of the 25 practices that recruited less than two patients in all of the four treatment arms and is unlikely to give robust results unless practices recruited a much larger number of patients in each group.

Estimating the EVPI or the probability that treatment is cost-effective following regression analyses on costs and QALYs is challenging for trials with more than two treatment arms. This is because it is necessary to allow for correlations between costs and effects for up to eight treatment arms that are correlated due to being extrapolated using the same set of UKPDS-OM2 risk equation parameters. In the base case analysis, we addressed this challenge by repeating all regression analyses on each bootstrap, although this approach cannot easily be combined with the mixed model.

In principle, Monte Carlo simulation could be used after a mixed model to estimate the mean costs, QALYs and NMB for each treatment arm, and to estimate SEs around these figures and generate cost-effectiveness acceptability curves and the EVPI. Seemingly-unrelated regression could be used to allow for correlations between costs and QALYs. Monte Carlo simulation could then be used to randomly generate values for each regression coefficient, assuming parametric distributions and allowing for correlations between regression coefficients. The results could be used to estimate cost-effectiveness acceptability curves and the EVPI. We did not use this approach in the base case analysis because some GP practices recruited only two patients, which made it very difficult to get the mixed models with seemingly unrelated regression to converge and give reliable results.

In this case, only two treatment arms had a non-zero chance of having highest NMB at a £20,000/QALY ceiling ratio, which enabled us to use a simpler approach to estimate the probability of atorvastatin monotherapy being best value for money. For the purposes of this sensitivity analysis, we therefore calculated the probability of each treatment being cost-effective as the one-sided p-value on the treatment coefficient.[9] For Analyses 1 and 2, the treatment groups were coded as one dummy variable for each treatment other than atorvastatin alone, such that regression predictions were the same as the base case analysis, but all coefficients (and p-values) gave the difference between each treatment group and atorvastatin-only; the probability that atorvastatin alone had the highest NMB therefore equalled 0.5 times the largest p-value (since only the treatment with the largest p-value had a non-zero chance of being cost-effective in this study). For Analysis 3, treatments were coded up in same way as the base case analysis (see equation (3)) and the probability that treatment was cost-effective compared with no treatment equalled 0.5 multiplied by the two-sided p-value for that treatment.

This analysis give virtually identical results to the base case analysis, although the standard errors around the simple effects on net benefit for atorvastatin versus no treatment were a few pounds larger in the mixed model compared with the base case analysis. The random effects on GP practice

were very small (<0.0001) in all three analyses and subject to a very large amount of uncertainty: for example, in the base case analysis, the random effect was estimated as 0.0000278 (SE: 0.0085854) - probably as some GP practices recruited only two patients.

References for Appendix 3

1. Diabetes UK. Diabetes in the UK 2010: Key statistics on diabetes. March 2010 https://www.diabetes.org.uk/resources-s3/2017-11/diabetes_in_the_uk_2010.pdf. Accessed 4 December 2019.

2. Scottish Intercollegiate Guidelines Network (SIGN). Management of diabetes costing template. May 2010 https://www.sign.ac.uk/sign-116-and-154-diabetes.html. Accessed 4 December 2019.

3. National Institute for Health and Care Excellence. Guide to the methods of technology appraisal 2013. 2013 https://www.nice.org.uk/process/pmg9/chapter/foreword. Accessed 16 April 2020.

4. Dakin H, Gray A. Economic evaluation of factorial randomised controlled trials: challenges, methods and recommendations. *Stat Med* 2017;36:2814-30.

5. Dakin H, Leal J, Briggs A, et al. Handling uncertainty when using patient-level simulation models to extrapolate clinical trial data. *Med Decis Making (in press)* 2020.

6. Montgomery AA, Peters TJ, Little P. Design, analysis and presentation of factorial randomised controlled trials. *BMC Med Res Methodol* 2003;3:26.

7. Gomes M, Grieve R, Nixon R, et al. Methods for covariate adjustment in cost-effectiveness analysis that use cluster randomised trials. *Health Econ* 2012;21:1101-18.

8. Gomes M, Ng ES, Grieve R, et al. Developing appropriate methods for cost-effectiveness analysis of cluster randomized trials. *Med Decis Making* 2012;32:350-61.

9. Hoch JS, Briggs AH, Willan AR. Something old, something new, something borrowed, something blue: a framework for the marriage of health econometrics and cost-effectiveness analysis. *Health Econ* 2002;11:415-30.
